# Query by Example: Semantic Traffic Scene Retrieval Using LLM-Based Scene Graph Representation

**DOI:** 10.3390/s25082546

**Published:** 2025-04-17

**Authors:** Yafu Tian, Alexander Carballo, Ruifeng Li, Simon Thompson, Kazuya Takeda

**Affiliations:** 1 Graduate School of Informatics, Nagoya University, Furo-cho, Chikusa-ku, Nagoya 464-8603, Japan; 2 State Key Laboratory of Robotic and Intelligent System, Harbin Institute of Technology, Harbin 150000, China; lrf100@hit.edu.cn; 3 Tier IV Inc., Nagoya University Open Innovation Center, 1-3, Mei-eki 1-chome, Nakamura-Ward, Nagoya 450-6610, Japan; alexander@g.sp.m.is.nagoya-u.ac.jp (A.C.); simon.thompson@tier4.jp (S.T.); 4 Faculty of Engineering, Gifu University, 1-1 Yanagido, Gifu City 501-1193, Japan; 5 Institute of Innovation for Future Society, Nagoya University, Furo-cho, Chikusa-ku, Nagoya 464-8601, Japan

**Keywords:** traffic scene retrieval, visual LLMs, subgraph isomorphism matching, scene graph, query by example

## Abstract

In autonomous driving, retrieving a specific traffic scene in huge datasets is a significant challenge. Traditional scene retrieval methods struggle to cope with the semantic complexity and heterogeneity of traffic scenes and are unable to meet the variable needs of different users. This paper proposes “Query-by-Example”, a traffic scene retrieval approach based on Visual-Large Language Model (VLM)-generated Road Scene Graph (RSG) representation. Our method uses VLMs to generate structured scene graphs from video data, capturing high-level semantic attributes and detailed object relationships in traffic scenes. We introduce an extensible set of scene attributes and a graph-based scene description to quantify scene similarity. We also propose a RSG-LLM benchmark dataset containing 1000 traffic scenes, their corresponding natural language descriptions, and RSGs to evaluate the performance of LLMs in generating RSGs. Experiments show that our method can effectively retrieve semantically similar traffic scenes from large databases, supporting various query formats, including natural language, images, video clips, rosbag, etc. Our method provides a comprehensive and flexible framework for traffic scene retrieval, promoting its application in autonomous driving systems.

## 1. Introduction

The rapid retrieval of a large number of traffic scenes is a significant challenge in the field of autonomous driving research. Currently, many autonomous driving companies and research institutes record thousands of hours of traffic scene data daily to build extensive traffic scene databases, which are used for testing and validating autonomous driving systems [1,2,3,4,5,6,7]. These databases contain a vast number of traffic scenarios; however, retrieving scenes similar to a specific query from these databases presents a considerable challenge.

The concept of “similarity” in traffic data differs from the usual notions of similarity in image or video data retrieval tasks. Depending on user needs, the definition of “similarity” may vary. For example, researchers focusing on low-level vehicle control algorithms may be more concerned with geometric and environmental information, such as vehicle position, speed, acceleration, road type, and weather. A notable example of this is SiaSearch [8] (now Scale Nucleus [9]), which allowed users to search specific scenes in datasets. By using a query system similar to SQL, the search system is able to locate the corresponding clips. For example, to find scenes in the nuScenes dataset under rainy conditions, with over three cars in view, while the ego-vehicle is in motion, the following SQL like query can be used: "dataset_name = ’nuScenes’ AND vehicle_following = ’True’ AND precip_type = ’RAIN’ AND forward_velocity >= 5 AND num_cars >= 3".

However, researchers in vehicle safety systems might be more interested in surrounding vehicles’ intent, such as whether nearby vehicles are about to change lanes or stop. Hence, a universal method is needed to retrieve scenes similar to a given query based on specific user requirements.

Another challenge in retrieving data is the heterogeneity of these datasets. Some datasets include advanced information like LiDAR and high-definition maps, while others, such as those focused on scenarios involving drunk driving or traffic accidents [3], only contain video data. Despite their importance and high cost, these datasets share one major characteristic as follows: they all contain video data (but do not include camera parameters, this omission makes it difficult to reconstruct the geometric information of individual elements from video alone). Thus, the best approach for traffic scene retrieval is to only accept video data as input.

Traditional video processing and retrieval methods [10,11,12,13,14,15] are difficult to apply to traffic scene datasets because most traffic scenes are highly similar, with differences mainly with regard to small details. On a higher level, the differences lie in vehicle and pedestrian intentions and road structure, while on a more detailed level, they involve geometric information such as object positions and speeds. Traditional video processing methods struggle to differentiate between this information and are often overwhelmed by irrelevant details such as weather, lighting conditions, building details, or advertisements.

In the above example using SQL-like query language, the user must be familiar with specific attribute names like “num_cars” to denote the number of vehicles. With the advent of large language models (LLM), it becomes possible to consider more human-friendly query approaches such as “Find in nuScenes dataset, clips which include 3 or more cars, rainy conditions, while the ego-vehicle is in motion”. Combining LLMs with vision (or VLMs), the user could provide an example image or a short video clip of the type of scene in mind, with the implicit meaning “Find scenes like this”. VLMs allow users to do query by example, which is the approach we propose in this research.

Therefore, a structured approach is needed to represent traffic scenes, one that preserves high-level information while ignoring irrelevant details. To this end, we propose a scene representation method based on topological graphs, known as the Road Scene Graph (RSG) [16,17,18], for traffic scene retrieval. Our method allows for example-based queries to retrieve scenes similar to the query from large traffic scene databases. It also supports various input formats, including natural language queries, images, video clips and even ROS/ROS2 [19,20] bag files from Autoware [21]. This is the unique aspect of our research. By integrating multimodal information (natural language, images, and videos) into a unified retrieval framework, we can achieve a more flexible retrieval method and support “Query by Example” retrieval. For example, when an autonomous driving system encounters a difficult scenario, it can query the database for scenes similar to the case in various dimensions to achieve a higher quality safety assessment.

**Figure 1:**
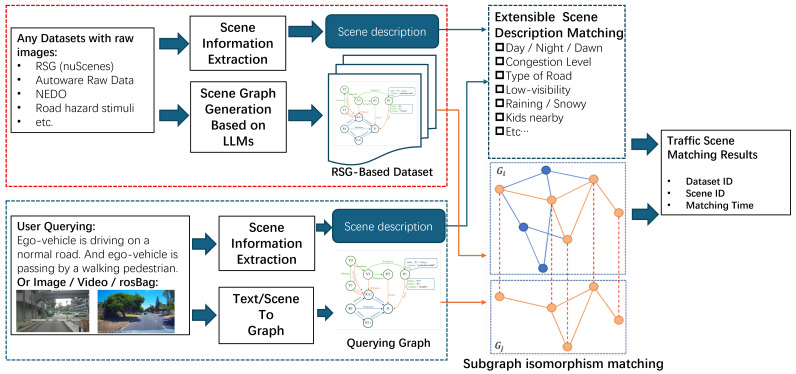
Schematic of our proposed method for traffic scene retrieval. There are three main components: RSG-LLM, to pre-generate RSG-based scene representations. Scene attribute predictor, to generate a set of scene attributes. Furthermore, the retrieval engine, which accepts various input formats and retrieves similar scenes from the database.

Previously, we implemented an algorithm that uses Graph Neural Networks (GNNs) to generate scene graphs based on object geometry [16]. However, this approach relied on high-precision bird’s eye view data, which required high-quality object geometry information. As a result, the method was difficult to apply to datasets outside of nuScenes. With the rapid development of Large Language Models (LLMs) and Visual LLMs in recent years, we see an opportunity to leverage Visual LLMs for generating RSGs. Visual LLMs can produce high-quality scene descriptions, and we believe they can also generate high-quality RSGs. We refer to this approach as RSG-LLM. As Figure 1 illustrates, first, RSG-LLM is used to generate RSG caches for traffic scenes in the database. These RSG caches are then used for traffic scene retrieval. Simultaneously, LLMs generate a series of traffic scene attributes, which are a set of enumerated properties such as weather, road type, and whether children are present nearby. By matching these attributes, we offer a more scalable traffic scene retrieval method.

However, constructing RSGs using VLMs is not an easy task. Many challenges are related to the engineering aspects, such as the limited length of visual tokens that LLMs can accept, causing keyframes to be missed when sampling from videos. Other challenges come from the models themselves, such as the lack of driving-related common sense in general LLMs, errors in visual symbol-to-language alignment (grounding), difficulties in handling contradictory visual scenes, and the negative impact of structured outputs on the quality of LLM outputs. This paper analyzes these errors and failures and proposes several improvement methods.

To better handle the complex context and dynamic interactions in scenes, in RSG-LLM, we leverage the object tracking capabilities of current multi-object tracking and the powerful generalization and scene understanding capabilities of Visual Language Models. To combine these two, we propose the LLM-Call-Graph tool, which aligns the results of object tracking with the output of LLM through multi-stage input, thereby generating more accurate RSGs.

We believe that our method can be applied to autonomous driving system scene retrieval. For example, when an autonomous driving system encounters a new scenario, similar scenarios can be retrieved from the database to better predict and plan actions. This is also a key method for implementing retrieval in Retrieval-Augmented Generation (RAG).

The main contributions of this paper are as follows:

1. We propose a scene representation method based on topological graphs (Road Scene Graph, RSG), and we implement an efficient traffic scene retrieval method based on this representation.
2. We analyze the errors and failures of LLMs in generating RSGs and propose several improvement methods.
3. We propose a benchmark RSG-LLM for applying LLMs to RSG generation. This benchmark contains 1000 traffic scenes, their corresponding natural language descriptions, and RSG topological graphs. These descriptions have been manually reviewed and corrected to ensure accuracy. The benchmark can be used to evaluate the quality of RSG generation by LLMs and provides a standard for future research.
4. We present a traffic scene retrieval method based on RSGs that can be applied to any dataset containing visual information without requiring camera parameters. In the query phase, the method accepts various input formats, such as natural language queries, image queries, and video clips. This retrieval method can serve as the Retrieval component in Retrieval-Augmented Generation (RAG), offering a new approach for integrating large language models with autonomous driving systems.
5. The RSG-LLM Benchmark, as well as the framework we used to manage VLM interaction, can be found at the following link: https://github.com/TianYafu/RSG-LLM/tree/main (accessed on 3 April 2025).

Compared with our previously proposed approach [18], the main contributions of this paper are as follows: (1) we introduce the RSG-LLM Benchmark as a baseline tool to evaluate the performance of LLMs in RSG generation; (2) we propose the LLM-Call-Graph framework, which leverages a multi-stage input strategy to align object tracking results with an LLM’s output, thereby producing more accurate RSGs; and (3) we provide a more comprehensive assessment of the challenges and issues encountered when applying VLMs to RSG generation, along with a series of improvements to address these problems.

This paper is structured as follows: in Section 2, we present the relevant related works and state of the art (SOTA), while in Section 3, we discuss the details of our scene retrieval method. In Section 4, we present our RSG-LLM approach. In Section 5, we present the diverse evaluations to our RSG-LLM method, including a discussion of the errors induced by the VLMs. Finally, Section 6 lists the key take-aways of this work and future directions we are considering.

## 2. Related Work

### 2.1. Traffic Scene Retrieval

In recent years, traffic scene retrieval has become a hot research topic [16,22,23,24,25,26]. The core of this research field lies in the following two main issues: first, how to effectively represent traffic scenes, and second, how to define the criteria for the similarity between two traffic scenes. Unlike general image and video retrieval, traffic scene data are often highly similar, with differences mainly regarding minor details. Traditional image and video retrieval methods struggle to extract and differentiate key information, often being overwhelmed by irrelevant details such as weather, lighting, building details, or advertisements. Video search methods that perform well on general video datasets, such as MSVD (Microsoft Research Video Description Corpus) [10], Microsoft-VTT [11], and LSMDC [12,13,14,15] may not perform well on traffic scenes. Therefore, integrating prior knowledge about traffic scenes to filter out irrelevant details is crucial for traffic scene representation.

Video content retrieval aims to rapidly locate segments containing specific content or events within massive video collections. This task requires effective feature extraction and indexing methods. Traditional approaches rely on handcrafted visual features (e.g., SIFT), which fall under the classic content-based image retrieval (CBIR) paradigm. However, deep learning techniques have become the mainstream in recent years [27]. By leveraging convolutional neural networks (CNNs) and similar models to learn high-level feature representations from video frames, deep learning–based methods provide greater discriminative power and richer semantics compared to handcrafted features. For example, video systems may extract key frames and employ a pretrained CNN (e.g., ResNet) to obtain feature vectors, and they then fuse multi-frame information through temporal models (LSTM, 3D-CNN, etc.) to produce an embedding representation for each video clip. In terms of indexing, large-scale video repositories demand efficient search structures. A common approach is to build a vector index [28] that accelerates the matching of feature vectors; by clustering, quantizing, or hash-compressing video features, one can achieve millisecond-level retrieval with acceptable accuracy trade-offs.

Autonomous driving perception systems must detect accidents from driving videos and, ideally, predict potential accidents ahead of time (“accident anticipation”). In essence, this constitutes a specialized video analysis or a targeted event retrieval as follows: the system needs to “search” for the exact moment of an accident within real-time video feeds. Deep learning methods have been widely employed for accident detection and forecasting. Recent models achieve strong performance in identifying accidents, for instance, by localizing the time window and spatial location of collisions (i.e., which two vehicles come into contact).

Fang et al. [29] use a graph convolutional network to model the relationships among traffic participants (e.g., relative positions and speeds) and combine it with a Bayesian neural network to estimate predictive uncertainty, thus improving accident prediction accuracy while reducing false alarms.

Another difference is traffic scenes, which are commonly recorded using various sensors like multi-camera systems and LiDAR. For example, some datasets such as nuScenes [2] and the Waymo Open Dataset [1] include information captured by these sensors along with their intrinsic parameters. In contrast, other datasets like Road Hazard Stimuli [3] and KITTI [4] are recorded only by vehicle-mounted or stationary cameras, providing only video information. Some studies [5,6,7] have leveraged state-of-the-art (SOTA) visual models to directly retrieve data from images or video streams. These methods retrieve traffic scenes corresponding to user inputs by comparing text-to-video or image-to-video similarities. The advantage of these approaches lies in applying the latest advancements in computer vision. However, as previously mentioned, these methods may perform inadequately in retrieving certain attributes of traffic scenes while overlooking other significant attributes. In practical applications, users often need to retrieve these various attributes.

Another approach involves extracting one or more abstract representations from raw traffic scene data. For example, some studies focus on extreme weather conditions in traffic scenes, such as rain or snow, and use various sensors for scene classification. Others encode traffic scenes and define their similarities in the vector space [23].

Another method is to transform traffic scenes into natural language descriptions, followed by retrieving these descriptions [30]. For instance, using the driving state of the ego-vehicle to characterize and retrieve traffic scenes [24,25,26]. In our previous study [16,17], we focused on the objects surrounding the vehicle and their implicit relationships. We employed topological graphs to represent traffic scenes and used subgraph isomorphism matching for retrieval. However, the limitation of this method lies in the need for highly precise 3D bounding boxes and the corresponding map information to construct the topological graphs and align objects with the correct roads and lanes. These data are difficult to obtain in most datasets. Consequently, our current research focuses on directly generating topological graphs from video streams that can be used for matching.

### 2.2. LLM-Aided Research in Autonomous Driving

Since the introduction of large language models (LLMs) like GPT-4 [31], they have garnered widespread attention across various fields. Notably, multimodal LLMs integrate processing capabilities for image, LiDARs, and other modalities, enabling models to interact with their environment visually. This integration has sparked significant discussions about applying such models to the development of autonomous driving technology. Currently, research connecting LLMs and autonomous driving mainly focuses on data annotation, decision reasoning, and the development of multimodal end-to-end autonomous driving systems.

Many studies, such as PCA-EVAL [32], LMDrive [33], and DRIVEGPT4 [34], have introduced LLMs into autonomous driving not only because these models outperform existing state-of-the-art (SOTA) visual language models, but also because LLMs can provide a deeper analysis of the reasoning behind decisions, supporting decision making in complex scenarios. For example, the “Driving with LLMs” project [35] utilizes LLMs for the in-depth analysis and annotation of driving scenes, generating high-quality datasets to enhance the accuracy and reliability of autonomous driving systems.

Additionally, some projects are attempting to use LLMs to build closed-loop end-to-end autonomous driving systems [36], such as LMDrive [33], DRIVEGPT4 [34], and methods from Turing Inc. [37] input textual information about the objects surrounding the ego-vehicle into an LLM to predict the vehicle’s behavior and responses. Meanwhile, projects like LMDrive [33] explore enhancing the interaction between autonomous vehicles and humans through LLMs, including building systems capable of communicating with human drivers or passengers in natural language.

These studies highlight the potential of LLMs in autonomous driving technology, particularly in data processing, decision support, and human–machine interaction. As technology continues to evolve, LLMs are expected to play an increasingly significant role in the field of autonomous driving. To the best of our knowledge, this is the first work proposing to use LLMs, including VLMs, for traffic scene retrieval providing a natural language input, an image, or a short video clip to describe the type of scenes to search for.

## 3. Traffic Scene Representation Using RSG

To accomplish the task of traffic scene matching, as described earlier, a traffic scene segment (typically 4–6 s) is represented by the following two components:

- Extensible set of scene attributes: These are high-level, enumerate, descriptive features of the scene, such as weather conditions, road types, and driving behavior. This flexible approach captures various aspects of the scene, tailored to different user or application requirements. A list of extensible scene attributes is provided in Table 1.
- Scene description based on a topological graph (RSG): This structured representation captures the objects present in the scene and the relationships among them. The RSG allows for detailed and precise comparisons of scene dynamics.

By combining these two representations of traffic scenes, the similarity between two scenes can be quantified as a combination of their scene attribute matching and RSG structural similarity. This method provides a comprehensive and flexible framework for traffic scene retrieval, balancing both high-level attributes and detailed relational information.

**Figure 2:**
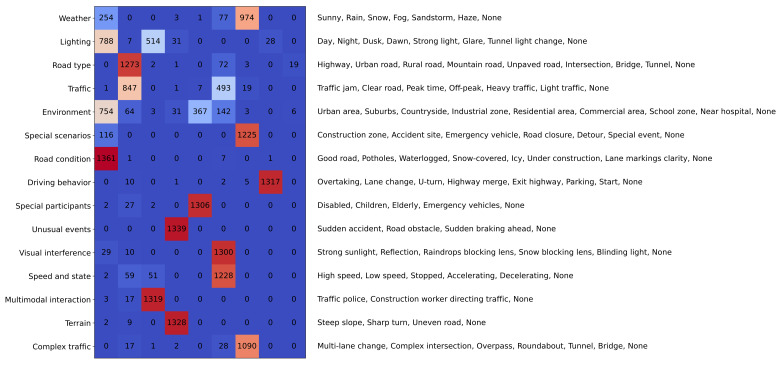
Distribution of scene attributes in the NEDO [38], nuScenes [2], and Road Hazard Stimuli datasets [39,40]. Each row represents a label type, and the values in each column represent the distribution of the corresponding labels in the dataset. To maintain the same dimension, we pad 0 at the end.

Extensible scene attributes: Figure 2 lists the set of extensible scene attributes we have defined. These attributes encompass various conditions that may occur in traffic scenes, such as weather, lighting, road types, traffic conditions, environment, special scenarios, and road conditions. Scene attributes can be extended based on users’ needs to adapt to different application scenarios. Users can select and weigh specific attributes according to their requirements, enabling customized and efficient scene retrieval. In practical applications, these attributes allow users to specify precise criteria for scene retrieval. For example, a user interested in congested traffic scenarios in urban areas at night can filter scenes based on the corresponding attributes.

We conducted scene attribute prediction on the following three datasets: NEDO [38], nuScenes [2], and Road Hazard Stimuli [39,40]. The distribution of attribute labels are shown in Figure 2. Although due to dataset limitations most traffic scenes are relatively homogeneous, this method can also detect many scenes that require special attention. In the NEDO dataset, for example, this includes scenes with children (27/1340 examples), construction workers directing traffic (a slightly more common scenario in the NEDO dataset, with 17/1340 examples), elderly, or disabled individuals (4/1340 examples), and so on.

**Figure 3:**
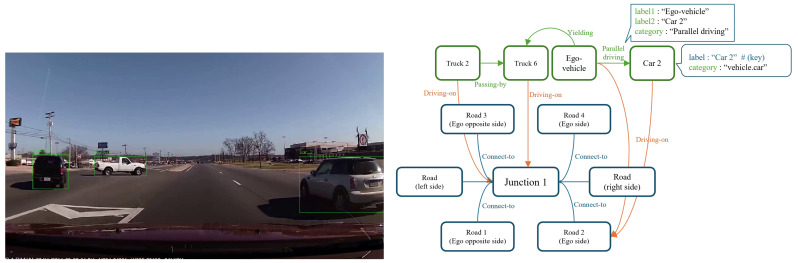
Simple schematic of the RSG, where the nodes of the graph, including vehicles, pedestrians, and map elements (roads, lanes, junctions on the bottom). The edges between these nodes represent the relationships among them.

**Figure 4:**
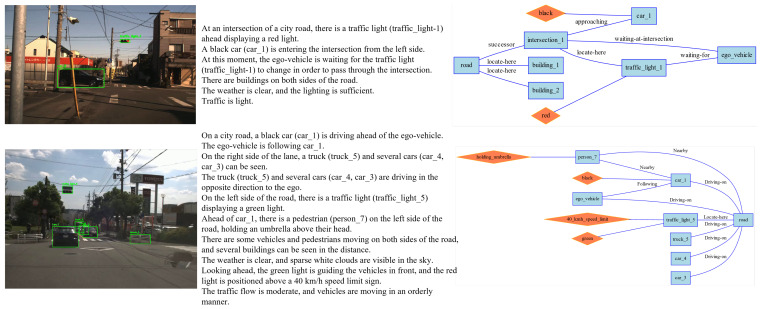
Examples of RSGs generated by RSG-LLM. Coming from a video clip and object detector, the RSGs capture high-level semantic attributes and detailed object relationships in traffic scenes. Furthermore, VLM can detect elements, such as persons with umbrellas, not found by the object detector.

Road Scene Graph (RSG): The RSG [16,17,18] is a directed graph where each node represents an object in the scene, and the edges denote relationships between these objects. A schematic of the RSG is illustrated in Figure 3, and a real-world example of the RSG is shown in Figure 4.

Table 2 provides examples of the node and edge relationships in the RSG. Although this table does not describe all relationships between objects, it covers the most common relationships between vehicles, pedestrians, barriers, and road elements. In the RSG, nodes represent traffic objects like vehicles, pedestrians, traffic signals, and so on. Furthermore, road components (such as roads, lanes, and junctions, typical of high definition road map formats such as ASAM openDrive [41]) are also described as nodes within the RSG. The relationships in RSG capture the interactions and spatial configurations between these objects, such as “vehicle driving on the road” or “pedestrian crossing the street”. Additionally, object attributes (e.g., vehicle color, type, and pedestrian characteristics) are represented as attribute nodes connected to the relevant object nodes. This structured representation not only captures the entities present in the scene but also describes the relationships between them, offering richer semantic descriptions compared to unstructured data.

## 4. RSG-LLM

In this section, we present a detailed methodology for generating Road Scene Graphs (RSGs) using a visual language model (VLM). Because RSG generation can be treated as a sequence generation task, we begin by introducing the structured representation of RSGs in Section 4.1, including the definitions of nodes, edges, and their interrelationships. We then explain the process of generating RSG-LLM, specifically describing how a VLM is utilized to produce a RSG. Next, in Section 4.2, we introduce the newly constructed RSG-LLM benchmark dataset, which serves as the ground truth for evaluating the accuracy of RSG-LLM generation and facilitates the subsequent algorithmic assessments. Finally, in Section 4.3, we illustrate how RSG-LLM can be applied to traffic scene retrieval, and how the RSGs it generates can be leveraged for that purpose. The retrieval process is divided into the following three stages: preprocessing the query input (Section 4.3.1), retrieval by natural language (Section 4.3.2), and retrieval by graph (Section 4.3.3).

### 4.1. RSG Generation Using Visual LLMs

Using Visual Large Language Models (VLMs) to generate Road Scene Graphs (RSGs) is essentially a process of converting raw visual information in images into structured text, i.e., generating a structured representation corresponding to the graph. As shown in Listing 1, the structured text (json) of the RSG contains parseable nodes, edges, and attribute information, requiring semantic correctness and accurate labeling.

**Listing 1.** Example of a structured text output representing a RSG.

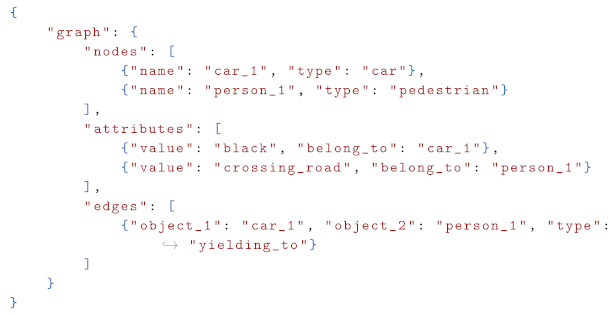



When modeling traffic scenes using relationships, we may use many objects and relationships, ranging from simple and common relationships, such as “vehicle driving on the road” and “vehicle following another vehicle”, to complex relationships, such as “truck overturned on the road” and “vehicle approaching a cat crossing the street”. In our previous research [17], we used graph neural networks to predict fixed relationship types in a scene. The predicted label set was fixed and expert-designed. In contrast to the aforementioned method, which extends from predicting fixed labels to describing scenes in a general manner, the RSG generated by LLMs can predict a broader range of few-shot relationship categories. However, this also introduces a trade-off. Fixed label sets are easy for users to understand and use, but they sacrifice the model’s generalization ability. Conversely, RSGs generated by LLMs can predict a broader range of few-shot relationship categories, but users may require more time to understand and use them. In the post-processing phase, we can aggregate or exclude labels based on predefined categories to reduce the complexity of the RSG. For the same object or relationship, LLMs may have multiple representations. For example, pedestrians may be represented as “pedestrian”, “people”, “kid”, “adult”, etc. In the post-processing phase, we use t-SNE-based clustering to group similar labels together and exclude uncommon labels.

While utilizing VLMs to generate such structured texts may seem straightforward, limitations of VLMs can result in semantic errors, incorrect labels, or missing information. Research [42] has pointed out that the reasoning ability of LLMs significantly declines under strict formatting constraints.

**Figure 5:**
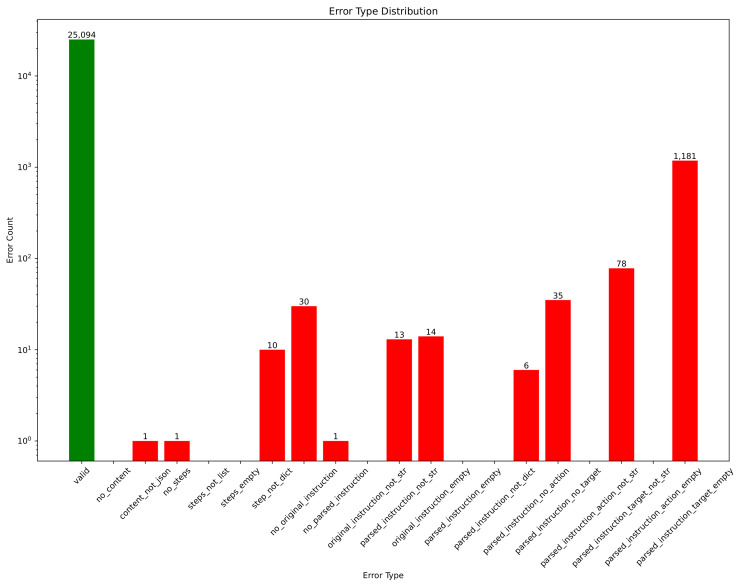
Performance variation of LLMs in generating instructions under different formatting constraints. The accuracy of LLMs decreases significantly as the depth (complexity) of the JSON format increases.

As an example to demonstrate the decline in LLMs’ reasoning ability under format constraints, in another study of ours, we attempted to convert instructions for an autonomous vehicle into a sequence of steps, where each step contained a target and an action, transforming a long instruction into an instruction tree with a certain depth. We used a parser to evaluate whether the LLM-generated instructions were valid, i.e., whether they conformed to predefined template requirements, and we analyzed the causes of the errors. As shown in Figure 5, as the complexity of the JSON format increases (with deeper node structures), the error rate of the LLMs also rises. This suggests that LLMs are highly influenced by formatting when generating structured text, and this effect intensifies as format complexity increases. As RSG generation involves a strict structured text output, LLMs are likely to face similar challenges. Therefore, we need an approach that addresses this issue.

A solution proposed by Ref. [42] employs a two-step "Natural Language to Format" (NL-to-Format) approach. Specifically, LLMs first respond in natural language, and the answer is subsequently converted into the target format. This method decouples the reasoning process from format adherence, thereby improving performance. Significant accuracy improvements were observed in datasets like GSM8K (which contains mathematical problems in natural language contexts) [43], Last Letter Concatenation (a task that involves concatenating the last letter of words) [44], and Shuffled Objects (an object tracking task) [45]. This demonstrates that separating reasoning from formatted output can enhance the performance of LLMs.

For the task of generating RSGs, we can adopt a similar two-step approach. First, LLMs describe the RSG in natural language, and then, this description is converted into the target structured format. This method can improve the accuracy of RSG generation, thereby enhancing the effectiveness of traffic scene retrieval.

**Figure 6:**
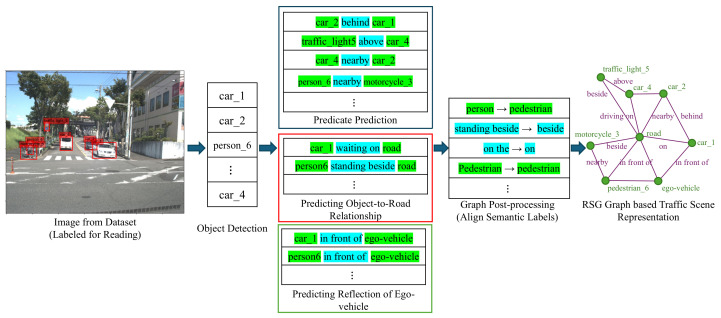
Two-step process for generating a RSG from visual input.

A two-step process for generating a Road Scene Graph (RSG) from visual input is shown in Figure 6. First, object detection is performed on the scene image, identifying the traffic elements present, such as vehicles, pedestrians, and traffic lights. Next, the visual large language model predicts predicates for the detected objects, inferring their relative positions and relationships, such as “vehicle 2 is behind vehicle 1” or “pedestrian 6 is near motorcycle 3”. Simultaneously, the model predicts object-to-road relationships, such as “vehicle 1 is waiting on the road” and “pedestrian 6 is standing beside the road”. In the post-processing phase, the semantic labels of the graph are aligned and optimized to ensure consistency. The final RSG represents the traffic elements in the scene and their interactions, such as the relative positions and behaviors of pedestrians, vehicles, and roads.

In traffic scenes, object interactions tend to be more complex than in general visual scenarios. For example, at an intersection with numerous vehicles or in a traffic-accident scenario, large numbers of similar vehicles often engage in complex interactions. Therefore, we integrate complex context and object interactions in traffic scenes at the following three levels:

First, when identifying objects, we use an object tracker to track them over time and assign each object in the scene a unique instance token, thereby consolidating interactions across different frames. For instance, consider a scenario where the ego-vehicle (ev) is following car1, which in turn is following car2. A vehicle to the right of ev, car3, continuously changes lanes to the left, passing between car1 and car2, then collides with the central divider, and subsequently veers right into car2. From the ego-vehicle’s perspective, car3 may be briefly invisible during its move between car1 and car2. However, by using an object tracker, we can continuously track car3’s motion, enabling us to associate the vehicle that changes lanes from the left with the same one that later collides with the divider and then with car2.

On top of object recognition, we employ segmented LLM calls to capture scene-level object context. For example, in one LLM call, the model focuses on describing the positions of objects in the scene from the “ego-vehicle” perspective, akin to a road user’s viewpoint. We fine-tune our LLMs to achieve this function. Then, in a second call, we feed the object-position descriptions, along with the visual information of the scene, back into the model to enhance its overall understanding. Descriptions from a traffic participant’s viewpoint often differ substantially from those grounded purely in the image. For instance, the original LLM output might be “car1 and car2 are on the left side of the scene; car3 is in front of car1; car5 and car6 are on the right”, whereas a specialized, fine-tuned LLM might say “car1 and car2 are at the left side of the intersection, with car2 positioned in the left-turn lane; car3 is on the opposite side of the intersection facing the ego-vehicle; car5 and car6 are on the right side of the intersection in sequence”. This type of description better reflects the perspective of a traffic participant and is more conducive to subsequent traffic-scene analysis.

At the feature-fusion layer, we use a VLM capable of processing video data. Built upon a large Transformer-based model, GPT-4o merges visual contextual modalities (in this case, video) with textual modalities. GPT-4o first employs a vision transformer (similar to ViT) to extract video features, and it then introduces cross-modal attention at intermediate layers so that language and visual features can interact repeatedly throughout the multi-layer Transformer. This process yields more accurate descriptions. According to the GPT-4o system card published by OpenAI, its training data include extensive amounts of images, audio, and video, allowing the model to interpret visual action sequences, language patterns, and audio details in real-world contexts. Consequently, it can align information across different modalities into a unified representation space, enabling joint multimodal reasoning.

It is important to note that, unlike traditional scene graph generation tasks, the process of generating RSGs involves two unique aspects. First, the relationship between the ego-vehicle and the objects in the scene must be determined. Although the ego-vehicle does not typically appear directly in images captured by the vehicle’s on-board cameras, it plays a central role in scene understanding. Therefore, VLM shall predict the relationships between the ego-vehicle and surrounding objects. Second, special attention is required for the relationship between objects and the road. The road is treated as a special object, and its relationships with other objects are unique. Unlike traditional object detection models, we do not specifically train the model to label the road. Instead, while detecting all objects in the environment, we predict their relationships relative to the road.

Considering a more generalized approach, we propose the LLM Call Graph to address this issue. By abstracting the calls to LLMs, we construct the LLM Call Graph as a Directed Acyclic Graph (DAG) $G_{\mathrm{LLMs}}=\left(V,E\right)$, where the following holds:

- $V=\{v_{1},v_{2},\ldots ,v_{n}\}$ represents the set of nodes in the graph, where each node $v_{i}$ denotes a single LLM call. For example, VLM receives a text description and outputs a structured json text that represents the RSG.
- E⊆V×V represents the set of directed edges between nodes, where an edge $e_{ij}=\left(v_{i},v_{j}\right)$ indicates that information flows from node $v_{i}$ to node $v_{j}$. For example, the graph generator receives the scene description, ego-vehicle-to-object relationship, and (optional) local traffic rules, and so on. Then, it puts them together as LLM input to generate the RSG.

Each node $v_{i}$ in the LLM Call Graph involves the following three input components:

1. System Prompt: Denoted by $P_{s}^{i}$, this represents the task to be executed and the output format required for the reasoning process at node $v_{i}$. Note that $P_{s}^{i}$ is fixed and does not change with the input data.
2. Variable Prompt: Denoted by $P_{v}^{i}$, this represents the specific input content for the reasoning process at node $v_{i}$, including variable data such as images and text.
3. Connection rules: Represented by the function $f_{i}$, the connection rules define how the input $P_{v}^{i}$ of node $v_{i}$ is derived from the outputs of its predecessor nodes.

For each node $v_{i}$, if its predecessor node set is $\mathrm{Pred}\left(v_{i}\right)=\left\{v_{p_{1}},v_{p_{2}},\ldots ,v_{p_{k}}\right\}$, and the generation set of those nodes is $\mathrm{Gen}\left(v_{i}\right)=\left\{o_{p_{1}},o_{p_{2}},\ldots ,o_{p_{k}}\right\}$, then a connection function $f_{i}$ is defined. The inputs of $f_{i}$ are the outputs from all predecessor nodes $\left\{o_{p_{1}},o_{p_{2}},\ldots ,o_{p_{k}}\right\}$, and its output is the variable prompt $P_{v}^{i}$ for the current node, as shown in Equation (1).

(1) $$P_{v}^{i}=f_{i}\left(o_{p_{1}},o_{p_{2}},\ldots ,o_{p_{k}}\right)$$

where $o_{p_{j}}$ is the output of predecessor node $v_{p_{j}}$. The connection function $f_{i}$ combines the outputs of all predecessor nodes according to specific rules, using them as the input $P_{v}^{i}$ for the current node $v_{i}$.

By defining this LLM Call Graph, we simplify the implementation steps for complex LLM systems, systematize the design of intricate calling processes, and automate the tracking of LLM inference flows. Through the predefined connection function for each node, we can automatically combine the outputs of predecessor nodes, thereby simplifying the implementation process of complex LLM systems. Therefore, the LLM Call Graph $G_{\mathrm{LLMs}}$ provides a general framework for handling complex LLM calls, improving inference quality and offering flexibility in system design.

**Figure 7:**
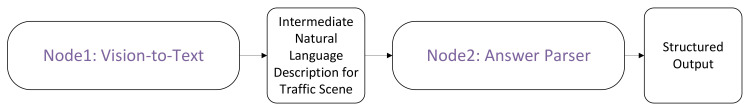
Sample of the two-stage RSG generation pipeline based on the LLM Call Graph (the detailed implementation of each node is shown in Figure A1).

**Figure A1:**
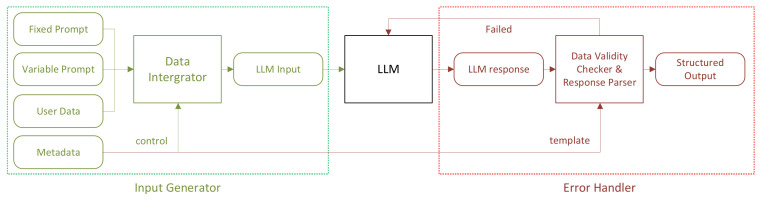
Implementation of a generic node in LLM Call Graph.

Based on the definition of LLM Call Graph, the two-stage graph generation method described earlier, can be represented in the form shown in Figure 7. (The detailed implementation of each node is shown in Figure A1). In this setup, the first node receives processed video data (post-object recognition) as input and provides a detailed description of the traffic scene in natural language, with minimal constraints. The second node takes the output from the first node as input and converts it into a highly structured JSON format corresponding to the RSG. In this way, we can separate the reasoning process of LLMs from the structured output, thus improving its performance.

**Figure 8:**
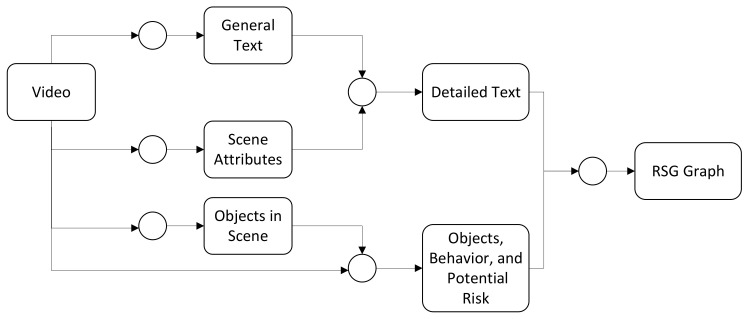
More complex example of the RSG generation pipeline, involving multiple LLM calls and information integration.

Figure 8 shows an example of a more complex LLM Call Graph. In this graph, video data is input into multiple LLM calls, which separately predict textual descriptions, scene attributes, object behavior in the scene, and the relationship of objects relative to the ego-vehicle. Then, a node integrates all this information and generates the corresponding RSG. This approach allows us to break down the LLMs reasoning process into multiple subtasks, thereby improving the performance of each subtask and enhancing the overall inference quality.

### 4.2. RSG-LLM Benchmark

To evaluate the performance of LLMs in generating RSGs, we propose a new benchmark dataset called the RSG-LLM Benchmark. This benchmark consists of data from three different sources as follows: NEDO [38], nuScenes [2], and Road Hazard Stimuli datasets [39,40]. The statistical information of these datasets is shown in Table 3.

The NEDO dataset contains 400 videos, each about 5–8 s long, and features unique Japanese traffic signs, left-hand driving scenarios, and typically narrow roads. Furthermore, the nuScenes dataset includes 300 videos, each 20 s long, but for consistency across datasets, we extracted 6 s video clips.

In practical applications, using a sliding window approach allows for near-real-time generation of RSGs for longer videos. Compared to the other two datasets, nuScenes provides highly accurate object location data. The Road Hazard Stimuli dataset includes 300 videos, each 7 s long, featuring a large number of traffic accident scenes as well as similar non-hazardous scenes. This dataset does not contain object geometry information, such as LiDAR point clouds, camera calibration parameters, or 3D bounding boxes. However, the traffic accident scenarios it contains are highly valuable and ethically difficult to reproduce. We believe that these three datasets are representative and provide a broad range of scenarios to comprehensively evaluate the performance of LLMs.

We introduce the RSG-LLM Benchmark to evaluate LLM performance in generating RSGs. It combines three datasets with diverse driving conditions as follows: NEDO [38] (400 short videos on narrow Japanese roads), nuScenes [2] (300 videos with high-accuracy object data, clipped to 6 s each), and Road Hazard Stimuli [39,40] (300 videos featuring both accident and non-accident scenarios). While NEDO and Road Hazard Stimuli lack 3D geometry, nuScenes provides precise object location information. Together, they offer a representative range of real-world scenes to comprehensively assess LLMs.

**Figure 9:**
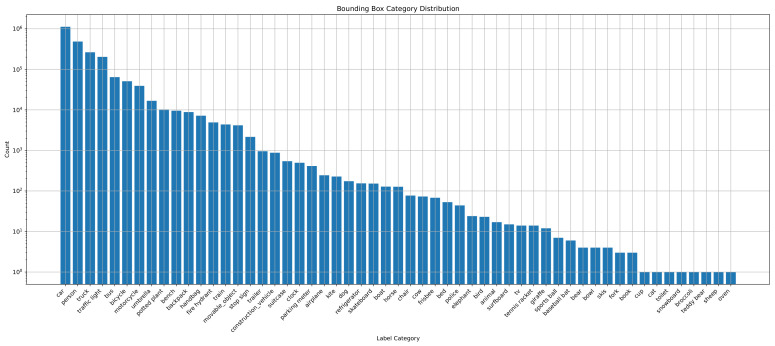
Bounding box category distribution in the RSG-LLM Benchmark dataset.

**Figure 10:**
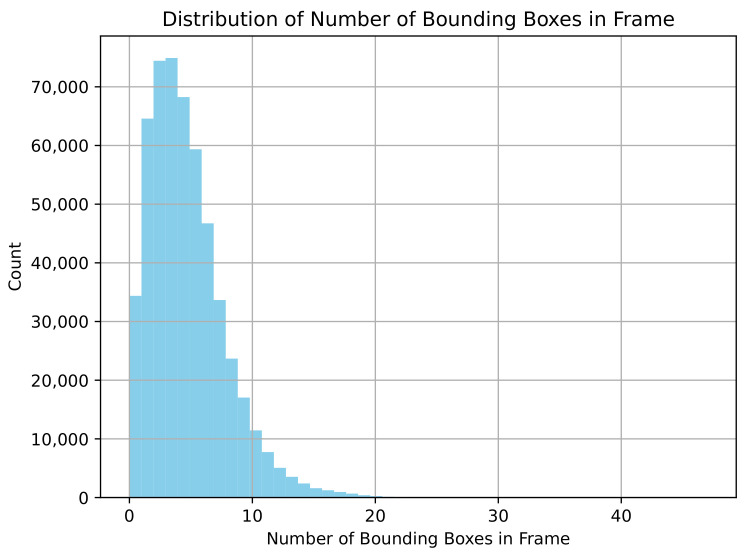
Bounding box amount distribution in the RSG-LLM Benchmark dataset.

**Figure 11:**
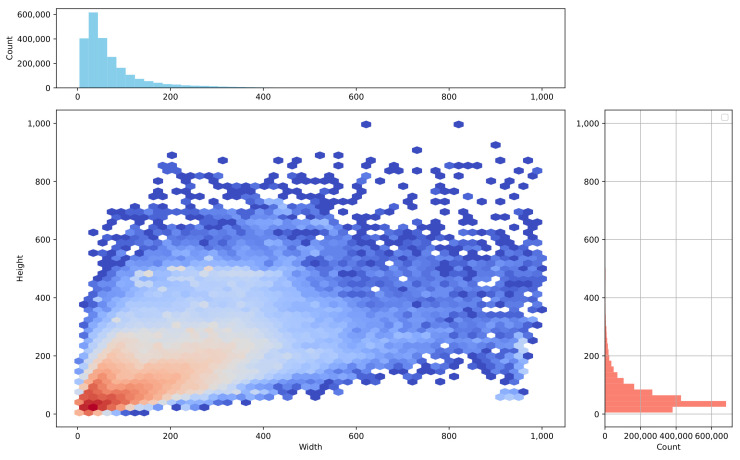
Bounding box size distribution in the RSG-LLM Benchmark dataset (width and height sizes are in pixels).

The RSG-LLM Benchmark dataset includes a wide distribution of labels. As shown in Figure 9, since these scenes are primarily recorded by vehicle-mounted cameras, labels such as vehicles, pedestrians, and traffic signals dominate the dataset. However, some long-tail labels, such as construction vehicles and animals, also account for a certain proportion. Figure 10 illustrates the distribution of the number of bounding boxes per frame. On average, each frame contains 4–5 bounding boxes, though some images contain more than 20 bounding boxes (most cases appear in nuScenes dataset). Figure 11 shows the distribution of bounding box sizes in the dataset, with most bounding box sizes ranging between 10 and 200 pixels. A few frames contain very large bounding boxes, such as when a truck passes the ego-vehicle and occupies the entire frame.

### 4.3. Traffic Scene Retrieval Based on Road Scene Graphs

Traffic scene matching based on RSGs is divided into two parts as follows: a topological graph matching method and a scene attribute-based matching algorithm. As described earlier in this study, a traffic scene segment (4–6 s) is represented by the following two parts: extensible scene description information and scene description based on a topological graph (RSG). Therefore, for any given scene, its matching score *s* with the user input is determined by a combination of the matching scores from these two parts, $s_{i}$ and $s_{g}$, where *i* stands for information and *g* for graph, respectively.

First, for the extensible scene description, we define an enumerated vector $I_{s}$, which includes a set of user-specified scene attributes, such as weather conditions, lighting, traffic congestion level, road conditions, and special events (e.g., the presence of children). The recognition of these features mainly relies on the VLMs as a Visual Question Answering (VQA) system. For each user query, we construct a query vector $I_{q}$ with the same dimensions as $I_{s}$, with each element corresponding to a specific attribute in $I_{s}$. Additionally, we introduce a query weight vector $W_{q}$, which has the same dimensions as $I_{s}$ and $I_{q}$, to represent the importance of different attributes. For example, when a user searches for all rainy weather traffic scenes, regardless of whether it is day or night, we assign values to the elements related to weather and time in $W_{q}$. Thus, the scene’s description information matching score $s_{i}$ with the user input is calculated in Equation (2), where ∘ is the element-wise comparison operation.

(2) $$s_{i}=\sum_{i=1}^{n}\left(I_{si}\circ I_{qi}\right)*W_{qi}$$

#### 4.3.1. Preprocessing Query Input

During the user search process, we allow users to input scenes (in the form of images) or use natural language commands to describe a scene. For image inputs, we process using the same method described in the previous section to generate the graph structure representing the scene. For natural language inputs, named entity recognition (NER) [46,47] and relation extraction are well-established tasks in the field of natural language processing, with relatively complete solutions. Since we have already constructed the related prompts, we use the same model for generating RSGs, calling, for instance, GPT-4o for inference. At this step, we convert the user’s natural language input into structured information equivalent to the RSG. Additionally, we provide a set of radio buttons for determining specific scene attributes, such as lighting, weather, road type, etc. This input is converted into the user search weight vector $W_{q}$, as described earlier.

#### 4.3.2. Query by Natural Language

Considering that when the input is natural language the user may not provide detailed descriptions of all scene attributes, during the preprocessing of the dataset, we extract all attributes in $I_{s}$. Therefore, the “element-wise comparison” operation ∘ in Equation (2) is unidirectional, meaning it only compares the elements in $I_{s}$ that appear in the user input $I_{q}$. The matching score for the topological graph, $s_{g}$, is binary. This means that either the query subgraph $G_{\mathrm{query}}$ matches a subgraph in the database $G_{i}$, or it match no subgraph in the database.

#### 4.3.3. Query by Graph

For topological graph matching, in this paper we adopt the VF2 [48,49] graph isomorphism-based matching algorithm. The subgraph isomorphism problem is a computational task where two graphs, $G_{1}$ and $G_{2}$, are given as input. The task is to determine whether $G_{1}$ contains a subgraph isomorphic to $G_{2}$. As shown in Algorithm 1, VF2 is a classic and widely used subgraph isomorphism algorithm that can determine whether two subgraphs are isomorphic. However, since the VF2 algorithm is an NP-complete problem with a high upper bound on time complexity (O(V!V)) and average time complexity (ranging between $O(V^{2})$ and O(V!V)), we need to control the size of the graph and convert it into an undirected graph to ensure the algorithm’s feasibility. Therefore, in practical applications, we limit the number of nodes in the graph to ensure the viability of the algorithm. Furthermore, the node and edge labels are used for pre-pruning graph while matching, making the matching of RSGs more efficient compared to general topological graphs.
**Algorithm 1:** Road scene graph matching algorithm M(s)
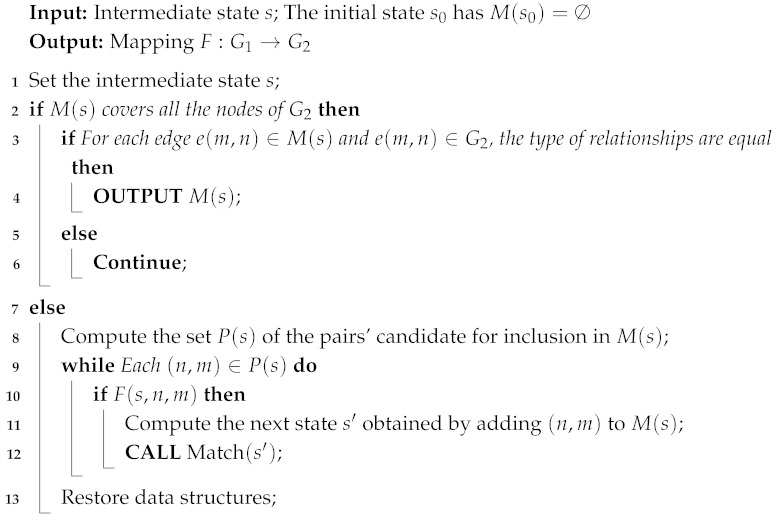


## 5. Experiment

### 5.1. Experiment Setup

As described in Section 4.2, we conducted experiments using a portion of the data from the NEDO [38], nuScenes [2], and Road Hazard Stimuli datasets [39,40] (1000 scene in total) and constructed the RSG-LLM Benchmark dataset. The data used for the experiments are shown in Table 3. For object recognition in the front end, we used the YOLO v8x [50] model for the identification and rendered the recognition results as prior information in the video. During inference, we used GPT-4o [31], the current best-performing visual language model (VLM), for reasoning and generating scene-related textual descriptions. Similarly, we used GPT-4o for generating RSGs.

**Figure 12:**
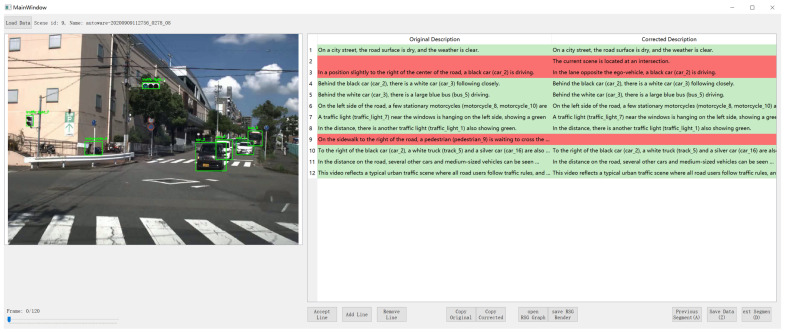
Annotator tool for original VLM output (**left**) and manual assertion verification (**right**). Furthermore, an example of assertion correction. Here, the completely correct assertions are shown in green, while red indicates incorrect assertions. The empty lines in the “Original Description” section indicate that these lines were missing in the assertions generated by the VLM and were manually added during the correction process. The “Corrected Description” section shows the assertions after manual correction. Detailed information (scene description and Natural Language Description) can be found in Figure 13 and Figure 14.

**Figure 13:**
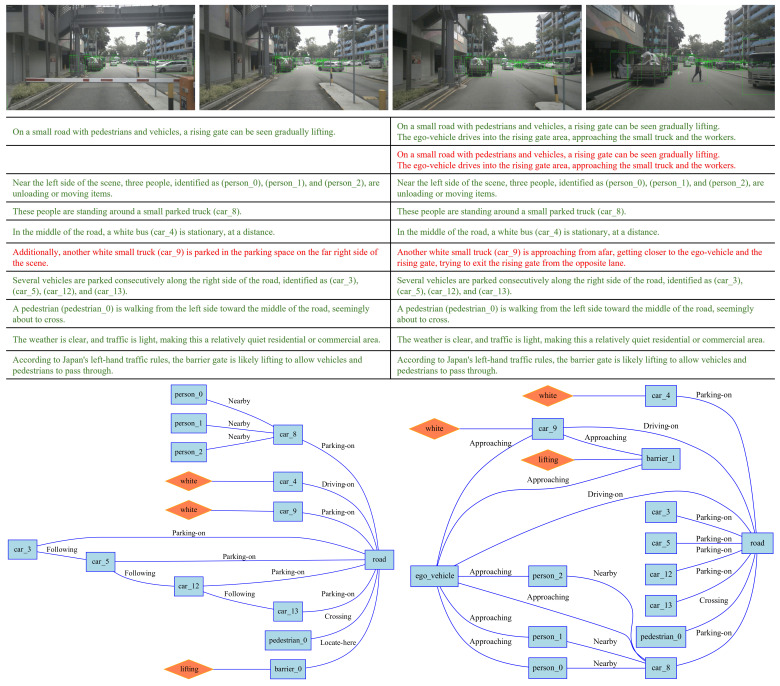
Example of our RSG generation pipeline. The top shows the images from the video. Furthermore, the middle left shows the original environment description generated by the VLM, while the environment description after manual correction is depicted on the right. Green lines indicate that the environment description was not modified, while red lines indicate modifications. The bottom shows the RSG corresponding to the environment description. This scene comes from the nuScenes dataset and shows an ego-vehicle passing through a rising gate and waiting for a pedestrian to pass.

**Figure 14:**
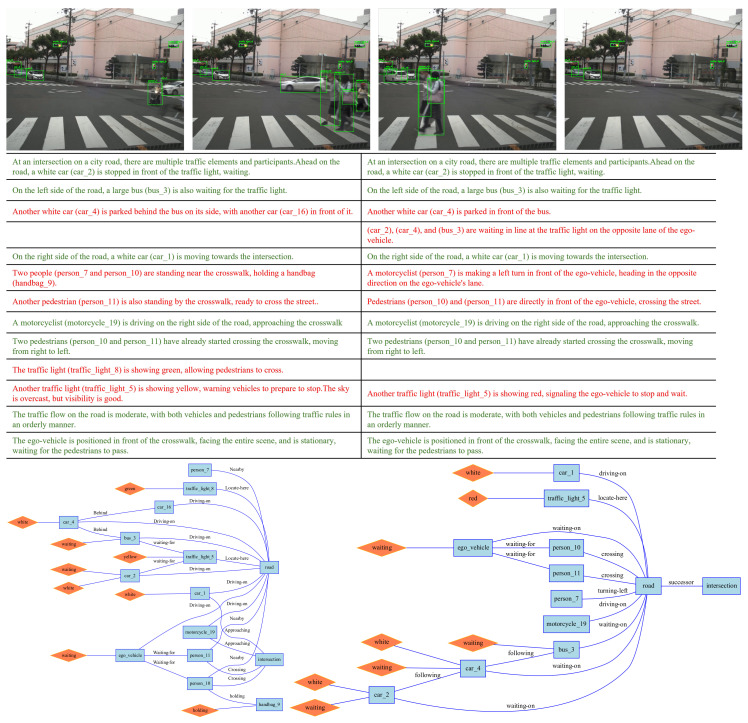
Another example of VLM-generated scene images. This scene comes from the NEDO dataset and shows a complex intersection with multiple pedestrians, vehicles, and traffic signals. The scene includes two pedestrians crossing the road, with vehicles waiting for them to pass.

We preprocessed the scene textual descriptions generated by the VLM, splitting them into lines, with each line corresponding to an assertion made by the VLM about the scene. Because the accuracy of textual description is of vital importance for the following RSG construction task. As part of the RSG construction, we manually verified the correctness of all assertions, corrected any errors, and added any missing assertions. The tool we used in this process is shown in Figure 12. When generating assertions, we require the VLM to generate labels for the corresponding objects, such as “person_4”. This creates a grounding from vision to text, allowing us to verify the correctness of the VLM’s object recognition during manual verification. Examples of modifications are shown in Figure 13 and Figure 14.

**Figure 15:**
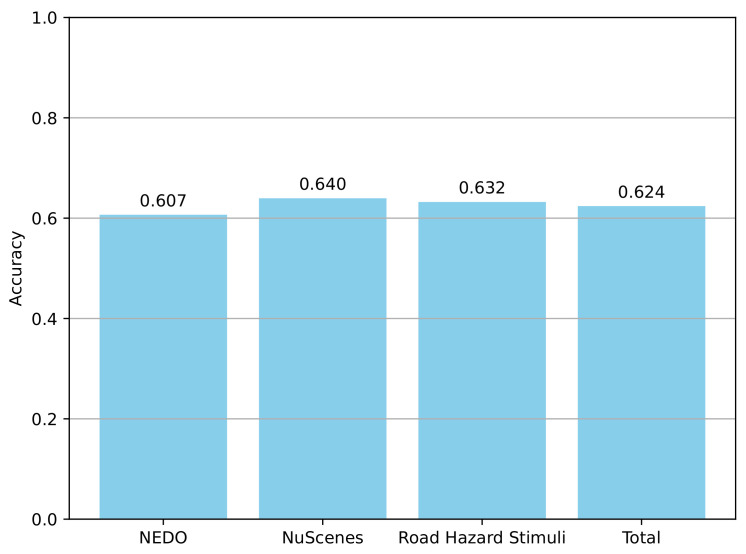
Assertion generation accuracy of the RSG-LLM method across three foundational datasets. Each dataset has its own unique characteristics, resulting in varying levels of accuracy. In the NEDO dataset, accuracy is lower due to relatively primitive label-rendering techniques. The Road Hazard Stimuli dataset also exhibits lower accuracy because it contains many brief, dynamic scenarios. By contrast, the nuScenes dataset achieves higher accuracy, owing to its more refined manual annotations and label rendering, although its larger number of objects and more complex inter-object relationships can also affect overall performance.

As shown in Figure 15, the overall accuracy of the assertions generated by GPT-4o is around 62%, but there are notable differences across the various datasets. During annotation, we observed that multiple factors—such as image quality, the number of frames sampled from the video, the parameters used for label rendering, the number of objects in the scene, and the overall complexity of object motion—can all influence accuracy. For instance, in the NEDO dataset, although there are fewer objects and simpler relationships, we used relatively low-quality label rendering (with thick fonts and bounding boxes), which lowered the accuracy. By contrast, for the NuScenes and Road Hazard Stimuli datasets—where object interactions are more numerous and complex—we adopted higher-quality label rendering (with optimized fonts and bounding boxes), thereby reducing errors introduced by label overlap. Even so, the accuracy for the Road Hazard Stimuli dataset remains relatively low, largely because it contains many brief, dynamic scenarios (particularly those involving traffic accidents). With fewer frames sampled, those scenarios become difficult to recognize, prompting us to devote significant effort to adding accident descriptions and revising assertions related to overall scene safety. Additionally, we sometimes merge the states of multiple objects into a single assertion (e.g., “car_1, car_2, car_3 are traveling in the oncoming lane relative to the ego-vehicle”), and if any one of these vehicles’ states does not match the assertion, the entire assertion is marked as incorrect—thus further contributing to the overall error rate.

Meanwhile, the emphasis in annotating these three datasets differs. In the NEDO dataset, which features fewer objects and a more constrained spatial environment, we place greater emphasis on describing each object’s state comprehensively. In the nuScenes dataset, which includes numerous objects and more complex interactions, the high-quality manual annotations can serve as a reliable tracking benchmark; however, the frequent overlaps among object labels increase the demands placed on the LLM’s scene understanding, thereby limiting accuracy in this dataset. Lastly, for the Road Hazard Stimuli dataset—which contains many short, dynamic sequences and traffic accidents—we focus more on capturing transient changes in the scene and on evaluating the LLM’s ability to identify accidents.

Figure 15 shows that GPT-4o’s generated assertions reach about 62% accuracy, overall, but vary among the datasets. Differences in the rendering quality, sampling rate, and object count affect the performance, for example, the NEDO dataset’s simpler environment had lower-quality label rendering, while the more complex nuScenes and Road Hazard Stimuli datasets benefited from better-rendered labels but still faced overlaps, motion complexity, and limited frames. Road Hazard Stimuli’s accuracy remained low due to the frequent brief accident scenarios, prompting extra effort to annotate crashes. Additionally, merging multiple objects into a single assertion increases the chance of errors. Each dataset’s annotation focus also varies. NEDO’s narrow scenes with fewer objects allow for thorough object-by-object descriptions, whereas nuScenes’ abundant objects and interactions demand stronger scene-understanding capabilities. Meanwhile, Road Hazard Stimuli emphasizes capturing accidents in short, dynamic sequences, testing the LLM’s ability to detect transient events.

**Figure 16:**
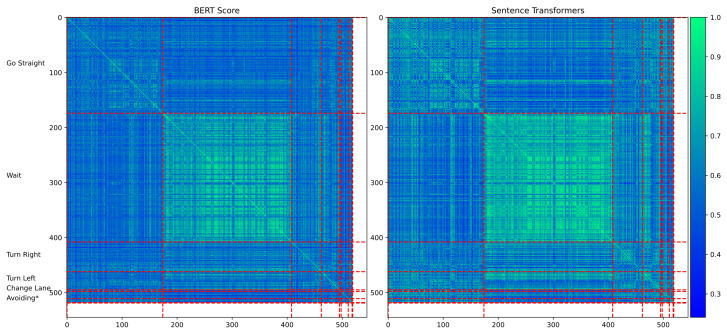
Similarity between textual descriptions calculated using BERT and sentence transformers. Most of the scene descriptions have high similarity and are difficult to distinguish. * The “Avoiding” label includes three sub-labels: “Avoiding Vehicles”, “Avoiding Pedestrians”, and “U-turn”. These have been combined for easier readability.

Since we have obtained these assertions, a natural idea is to use the similarity between assertions to perform traffic scene retrieval. However, as described earlier, unlike general image retrieval, most traffic scenes are actually quite similar, with important details being drowned out by a large number of irrelevant scene descriptions. As an example, we randomly selected 530 scenes from the NEDO dataset and manually classified them into eight categories based on the behavior of the ego-vehicle as follows:“Go straight”, “Wait”, “Turn Left”, “Turn Right”, “Change Lane”, “Avoiding Vehicle”, and “Avoiding Pedestrian”. We then calculated the similarity of the LLMs to the scene descriptions, clustered them based on similarity, and visualized them. We used two state-of-the-art methods, BERT [51] and sentence transformers [52], to calculate the similarity between the textual descriptions, as shown in Figure 16. Most of the scene descriptions have high similarity and are difficult to distinguish. As a qualitative analysis, the inter-class weighted similarity/intra-class weighted similarity ↓ (the smaller the better) is 0.9912:1.04. The Davies–Bouldin Index is used to measure the similarity, with scores ranging from 4.3 to 5.32 (the smaller the better, usually between [−1, −1]). This means that traditional text similarity calculation methods, such as BERT, cannot effectively distinguish between these scenes and strictly differentiate between scenes according to user input.

### 5.2. Traffic Scene Retrieval Based on Road Scene Graphs

**Figure 17:**
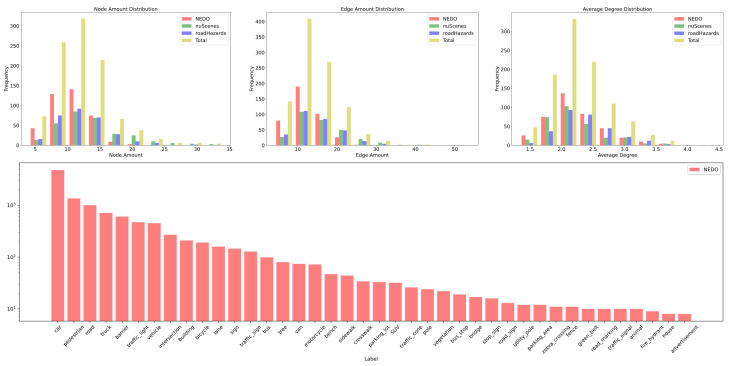
Statistics of the RSG-LLM Benchmark. The average number of nodes in the database (**top left**), the average number of edges (**top middle**), the average degree (**top right**), and the distribution of node labels (Top 20) (**bottom**).

We constructed RSGs based on all the annotated assertions in the RSG-LLM Benchmark. Examples of the actual topological graphs are shown in Figure 13 and Figure 14. To provide a more comprehensive understanding, for the graph data in the RSG-LLM Benchmark, Figure 17 shows part of statistical evaluation, including the average number of nodes in the database (Figure 17 (top left)), the average number of edges (Figure 17 (top middle)), the average degree (Figure 17 (top right)), and the distribution of node labels (Top 20) (Figure 17 (bottom)). As Figure 17 illustrates, although the dataset includes many object-dense scenes, the average number of nodes in the graph is about 12, with most having fewer than 35 nodes. This is because in the process of constructing the RSG, we used the VLM to exclude objects that are too far away to interact with the ego-vehicle and objects that cannot physically interact with the ego-vehicle. This allows us to reduce the size of the graph to improve matching efficiency. Considering that the time complexity of VF2 is related to the number of edges in the graph, the average degree of 2.3 and the average number of edges of 12 in the RSG-LLM Benchmark are very satisfactory results. This balances the exploration of semantic relationships between objects and matching efficiency.

**Figure 18:**
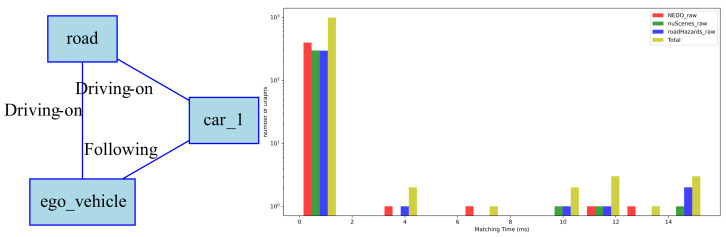
Matching time distribution of query “ego-vehicle follows another vehicle on the road” in the RSG-LLM Benchmark. The RSG topological graph of the query is shown on the left, and the query time distribution is shown on the right. The average query time is 2 ms/1000 scenes, with some queries taking up to 14 ms/1000 scenes.

**Figure 19:**
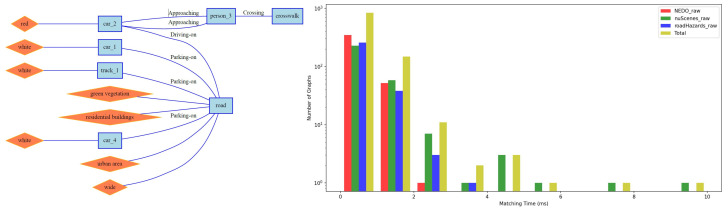
More complex query example. The query time distribution is similar to that of the simple query, indicating that our method is acceptable in terms of matching efficiency in the RSG-LLM Benchmark under normal driving scene complexity.

Consider a simple query “ego-vehicle follows another vehicle on the road”. The corresponding RSG topological graph is shown in Figure 18 (left), which is a graph with three nodes. The query time for this scene in the RSG-LLM Benchmark is shown in the histogram in Figure 18 (right). From the histogram, it can be seen that the query time for most queries is within 2 ms/1000 scenes due to the pruning strategy of VF2 terminating the search early. However, there are also some queries that take longer (14 ms/1000 scenes), which is acceptable for queries on 1000 scenes. Another more complex query example is shown in Figure 19. We used a more complex graph from the dataset to query the RSG-LLM Benchmark, and the query time distribution is similar to that of the simple query, indicating that our method is acceptable in terms of matching efficiency in the RSG-LLM Benchmark under normal driving scene complexity.

### 5.3. Error Analysis

#### 5.3.1. Error from Front-End Object Recognition and Label Rendering

**Figure 20:**
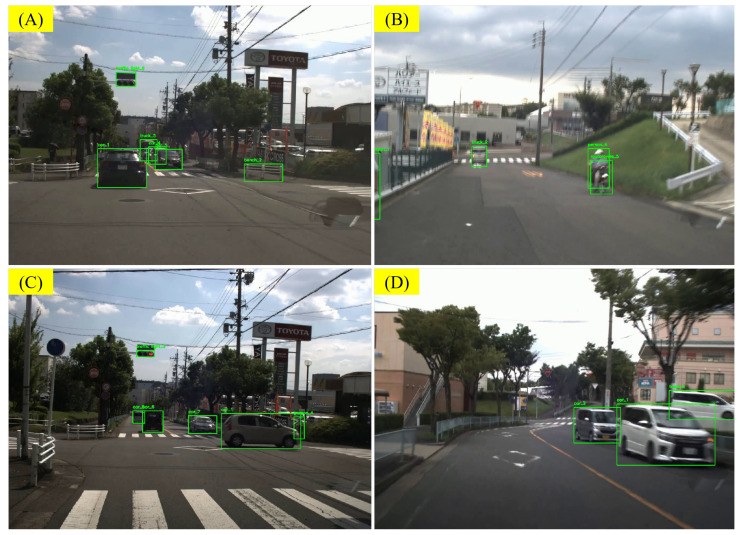
Typical error cases in the RSG-LLM Benchmark dataset. (**A**) YOLO misidentifies a Japanese traffic barrier as a bench. (**B**) YOLO recognizes a motorcycle and person4 as separate objects. (**C**) Object tracking introduces a non-existent vehicle. (**D**) Bounding box jitter causes the VLM to mistake parked cars for moving vehicles.

In the annotation phase, in addition to recording the accuracy of the assertions, we also recorded the reasons for high-frequency errors. One of the main sources of errors is the errors introduced by the object recognition model and label rendering process in the front end. For example, in some scenes (e.g., Figure 20A), YOLO [50] identified a typical Japanese traffic barrier as a bench. In other scenes, the front end recognized objects that do not actually exist. This can lead to false results and confusion during the VLM inference process. In another scene Figure 20B, YOLO recognized two independent objects, a motorcycle and person4, but did not identify them as a “rider”. What makes it worse is that the bounding box separates the motorcycle from the rider, making it difficult for the VLM to associate the two and correct the error (such corrections are rare, but they exist). This led to the generation of a non-existent pedestrian in the assertions generated by the VLM.

Additionally, errors can be introduced during the label and bounding box rendering process. In some cases, labels may be squeezed together and overlap, making it difficult for the VLM to recognize them, or they may obscure key objects. At low resolutions, label numbers may be misinterpreted, such as car5 being mistaken for car6 in very low resolution.

During object tracking, the motion of objects can cause label changing and flashing. In such cases, the VLM may introduce objects that do not actually exist in the scene. For example, in Figure 20C, due to the movement of vehicles, the VLM-generated assertions included a non-existent vehicle. Additionally, the results of object tracking may exhibit jitter, which can create an illusion of motion. As described in Figure 20D, the jitter of the bounding box caused the VLM to mistakenly believe that the vehicles car_1 and car_2 were moving. After removing the bounding box, the VLM was able to correctly determine that these two cars were parked on the side of the road.

During annotation, we also tracked common error sources. One major issue arises from front-end object recognition and label rendering. For example, YOLO [50] misidentified a Japanese traffic barrier as a bench in Figure 20A or detected nonexistent objects, confusing the VLM inference. In Figure 20B, YOLO labeled a motorcycle and person4 separately, instead of recognizing a single rider, causing the VLM to generate a spurious pedestrian. Labeling can introduce further errors if bounding boxes overlap or obscure key objects, especially at low resolutions (e.g., car5 is mistaken for car_6).

Additionally, object tracking can cause sudden label switches and jitter. In Figure 20C, vehicle movement led to an invented car in the VLM output, while jitter in Figure 20D made the VLM believe car_1 and car_2 were moving. Removing the bounding box then revealed they were actually parked.

**Figure 21:**
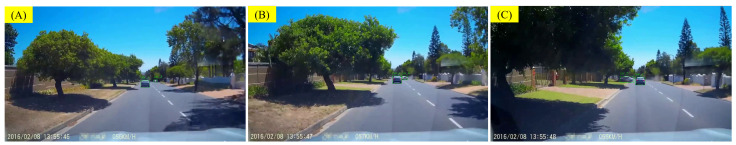
Another example of object tracking failure. (**A**), (**B**), and (**C**) sequentially illustrate the accident scenario. A distant vehicle repeatedly crosses in front of the vehicle ahead of the ego vehicle. Due to object tracking failure, the vehicle involved in the accident is incorrectly labeled as car_3, car_4, and car_5, in sequence. Furthermore, the VLM introduces non-existent objects into the image.

In the example shown in Figure 21, due to object tracking failure, the vehicle involved in the accident in the distance was initially labeled as car_3 at the beginning of the scene. Later, before being obscured by car_1, the vehicle was incorrectly labeled as car_4. Then, after being obscured by car_1, the vehicle was labeled as car_5 when the accident occurred. The VLM seems to have difficulty handling this error, leading to the introduction of non-existent objects and relationships in the image.

#### 5.3.2. Errors from Insufficient Frame Rate for Recognizing Sudden Accidents

**Figure 22:**
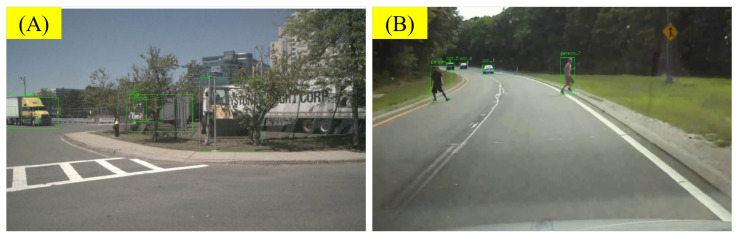
Errors from insufficient frame rate for recognizing sudden accidents. (**A**) The ego-vehicle makes a left turn in the last few frames of the video. (**B**) A pedestrian comes out in the last few seconds of the video.

Another type of error comes from the video processing. Due to the limited frame rate sampled from the video, the recognition of sudden events/accidents is insufficient. Current VLMs generally process videos as sequences of images. Although some VLMs claim to be able to generate high-quality annotations with a small number of sampled frames (e.g., a 50:1 ratio), this sampling rate is still insufficient for recognizing traffic scenes. In some scenes, this can lead to errors in the inference process. For example, in Figure 22A, the ego-vehicle makes a left turn in the last few frames of the video. However, due to the video sampling limitation, the VLM did not capture this information, leading to an inference error. In Figure 22B, a pedestrian comes out in the last few seconds of the video but is not correctly recognized by the VLM. This type of error is particularly common in traffic scenes, as sudden events in traffic scenes often occur in the last few seconds of the video. We believe that this is a problem that can be solved with the improvement of computing power and model capabilities. However, in current applications, this is a trade-off between cost and accuracy.

Due to low frame sampling, sudden events or accidents in the final seconds may be missed by the VLM. Improving sampling strategies or leveraging higher-frequency video sources could alleviate such omissions

#### 5.3.3. Analysis of Errors from VLM’s Illustration

**Figure 23:**
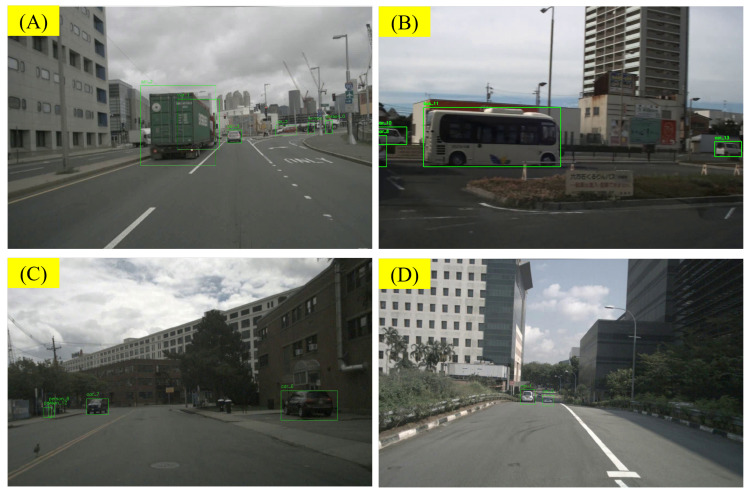
Difficulties for VLMs in judging the motion status of other objects in the scene. (**A**) The VLM has difficulty determining whether the truck car_2 is moving when the camera viewpoint is moving. (**B**) The VLM incorrectly judges that bus_11 is moving forward because the ego-vehicle itself is also in motion. (**C**) The VLM incorrectly confuses its own motion with the motion of surrounding objects. (**D**) The ego-vehicle has difficulty determining whether the vehicle directly ahead is moving or stopped.

For VLMs, it is also difficult to judge the motion status of other objects in the scene based solely on visual clues, especially when the ego-vehicle itself is in motion. As shown in Figure 23A, when the camera viewpoint is moving, the VLM has difficulty determining whether the truck, car_2, is moving, especially when there is a lack of clear reference points nearby. Similarly, in Figure 23B, because the ego-vehicle itself is in motion, the VLM incorrectly judges that bus_11 is moving forward. In Figure 23C, the VLM incorrectly confuses its own motion with the motion of surrounding objects, leading to the judgment that car_6 is moving forward. In Figure 23D, based solely on visual information, the ego-vehicle has difficulty determining whether the vehicle directly ahead is moving or stopped, especially on uneven roads like the one in the example. Additionally, due to the sampling process we used when processing the video, the LLMs have difficulty recognizing the presence of hazard lights. In many scenes, parked vehicles have their hazard lights on, but the VLM ignores this visual clue. In future research, we plan to use object kinematic information obtained from LiDAR or independent depth models as prior information and integrate it with the visual information. Thus, we aim to improve the VLM’s ability to judge the motion status of objects.

Determining an object’s motion from purely visual cues is challenging when the ego-vehicle itself is moving. In Figure 23A, limited reference points make it hard to tell if truck car_2 is moving. In Figure 23B, the VLM mistakenly thinks bus_11 is advancing due to the ego-vehicle’s own motion. Likewise, Figure 23C shows the model confusing its own movement with that of car_6. As seen in Figure 23D, the VLM also struggles to distinguish a stationary or moving vehicle on uneven roads. Moreover, sparse video sampling causes it to overlook hazard lights on parked vehicles. To address these issues, we plan to integrate object kinematic data from LiDAR or depth models, thereby enhancing the VLM’s ability to judge motion status.

**Figure 24:**
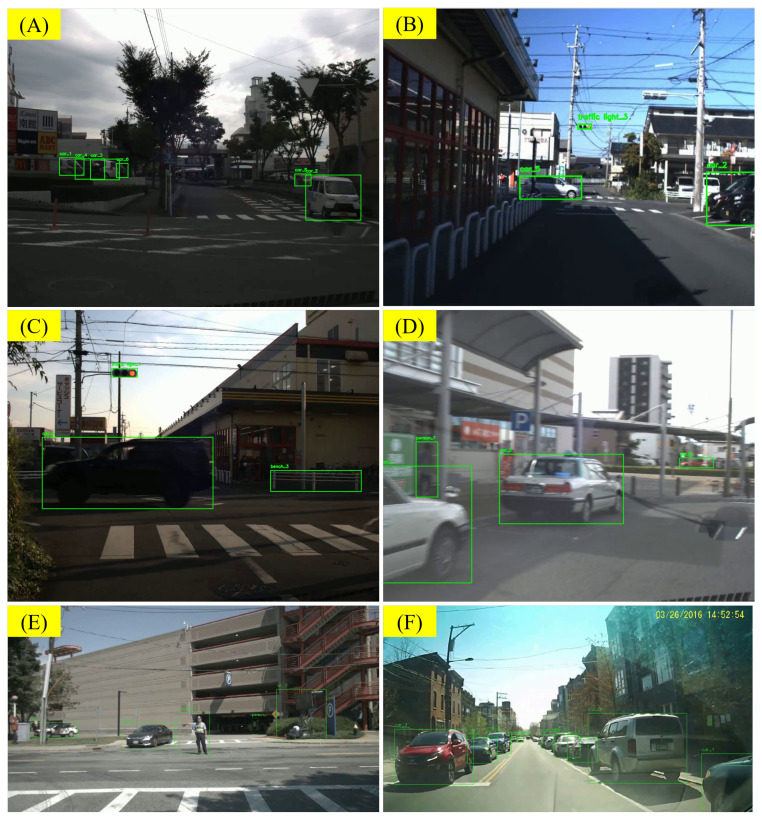
Errors in spatial relationship reasoning. (**A**) A car (car_5) is parked on the other side of the road, not near the van (car_2). (**B**) The direction and motion of car_5 is misjudged. (**C**) The direction of car_2 is misjudged. (**D**) The LLMs failed to recognize road divider and lane, so they mistakenly believe that car_3 and car_5 are parked in the same parking lot. (**E**) The black car (car_0) is supposed to be located behind the traffic director (person_0), not simply in their vicinity. (**F**) Car_2 is behind car_3, not in front.

Correctly determining the spatial position relationships of objects in the scene, especially the spatial position relationships in a traffic context, is a challenging task. During the manual correction process, we made numerous changes to the VLM’s descriptions of spatial position relationships to make them more precise. For example, “the vehicle is driving on the right side of the road” was corrected to “the vehicle is driving on the opposite lane to the ego-vehicle, heading towards the ego-vehicle”. However, there are still many problems that are difficult to solve. On the one hand, it is sometimes difficult to distinguish between “close” in the image and “close” in the spatial relationship. As shown in Figure 24A, there is a car (car_5) parked on the side of the road near the van (car_2). However, car_2 and car_5 are not directly related. On the other hand, due to motion blur and frame sampling, the direction of travel of car_2 is misjudged in Figure 24B. In Figure 24C, the LLMs were not aware of the existence of road dividers and lanes, so they mistakenly believed that car_3 and car_5 were parked in the same parking lot. In Figure 24D, the LLMs failed to recognize road dividers and lanes, so they mistakenly believed that car_3 and car_5 were parked in the same parking lot and were adjacent to each other.

Additionally, even if the spatial relationships between objects are correctly identified, converting them into spatial relationships in human descriptions is also challenging. In Figure 24E, behind the traffic director (person_0), there is a black car (car_0) in a parked state, waiting for the traffic director’s instructions. However, this “behind” is the reference object’s (person_0) behind, or the behind based on the road Frenet coordinate system. In spatial terms, car_0 is in front of person_0 rather than behind them. Similarly, in Figure 24F, car_2 is behind car_3, not in front of it. However, in some examples, the VLM may confuse spatial front and back with semantic front and back.

Determining spatial relationships is challenging in traffic contexts, requiring precise descriptions like changing “the vehicle is driving on the right side of the road” to “the vehicle is driving on the opposite lane to the ego-vehicle, heading towards the ego-vehicle”. Still, ambiguities persist. For instance, Figure 24A shows car_5 parked near car_2, yet they are not truly related. In Figure 24B, motion blur leads to an incorrect travel direction for car_2, while in Figure 24C,D, the LLMs fail to recognize road dividers or lanes, mistakenly grouping car_3 and car_5 together.

Even if object positions are detected, converting them into meaningful human descriptions can be confusing. For example, Figure 24E places car_0 behind person_0 in a Frenet sense, though visually it is in front. Similarly, Figure 24F shows that car_2 is actually behind car_3. The VLM may mix up spatial “front/back” with semantic “front/back”, causing further confusion.

#### 5.3.4. Error from Lack of Driving Common Sense

**Figure 25:**
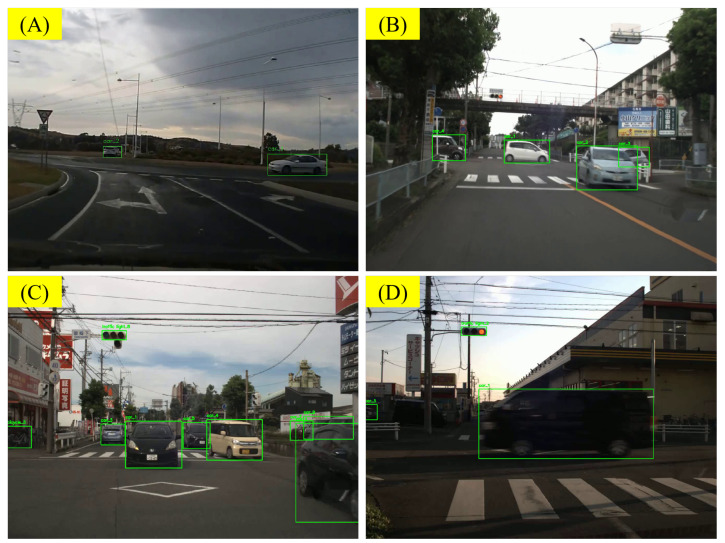
Errors from lack of driving common sense. (**A**) The scene is a roundabout, and the relationship between several vehicles is incorrectly judged. (**B**) The LLMs mistakenly believe that all vehicles need to wait at the intersection. (**C**) The LLMs cannot infer the color of the traffic signal from the traffic status. (**D**) The VLM mistakenly identified the red light as a yellow light.

In addition, existing general-purpose large language models have not been specifically optimized for traffic scenes. Therefore, in the inference process of VLMs and LLMs, some common sense about traffic scenes may be missing. What makes things more complex is that many traffic rules, cases, and traffic signs vary by region. Our RSG-LLM Benchmark dataset contains driving videos from multiple regions. For example, Japan and the United States are different (left-hand driving versus right-hand driving). Currently, it is impossible for us to fine-tune the visual capabilities of GPT-4o, but by providing VLMs with sufficient traffic rule information through appropriate prompting, this problem has been partially addressed. However, due to the complexity of traffic rules, some errors are still difficult to avoid. For example, in Figure 25A, the ego-vehicle is in a roundabout. Since the LLMs and our prompt do not define the traffic rules for roundabouts, the relationship between several vehicles is incorrectly judged in the output. In Figure 25B, the LLMs did not correctly judge the relationship between the red light and all vehicles at the intersection, so they mistakenly believed that all vehicles needed to wait. In Figure 25C, due to lighting conditions, it is difficult to determine the status of traffic lights. Unlike humans, the current LLMs cannot infer the color of the traffic signal from the traffic status of the vehicles at the intersection. In Figure 25D, in Japan, some traffic lights are more yellowish than the USA, so the VLM mistakenly identified the red light as a yellow light. Humans can infer that the current status should be a red light based on the traffic scene, but the VLM failed in this scene. In future research, we plan to dynamically introduce a traffic rule text as a VLM reference. Such a reference will be based on factors such as the region where the scene occurs and the type of scene. This will help improve the VLM’s reasoning ability in traffic scenes.

#### 5.3.5. Other Errors

**Figure 26:**
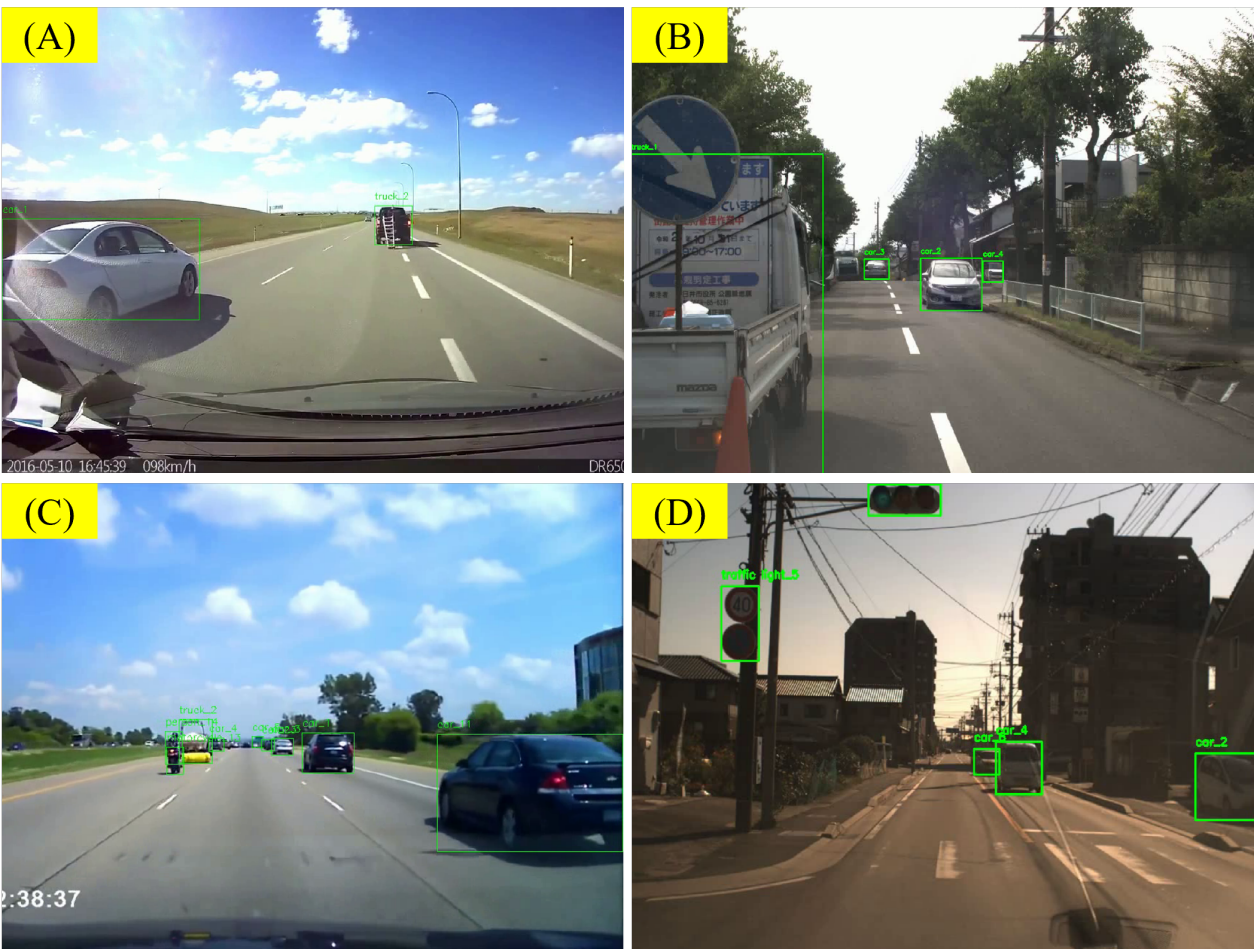
Other errors. (**A**) An object (a ladder) falls from a vehicle. (**B**) A truck is carrying a road sign, but the road sign is not in use. (**C**) Another sample of object falls. (**D**) A contradictory situation that the traffic light turns green, but the vehicle does not start.

In the previous sections, we discussed some of the most common errors made by VLMs in the field of traffic scene understanding. However, there are also some special or rare situations that can lead to errors by VLMs. For example, in Figure 26A, this is an error in which an object falls from a truck. However, the VLM did not correctly identify this situation and mistook the fallen ladder for another vehicle. In Figure 26B, a truck was carrying a road sign, but the road sign was not actually in use and misled the VLM. Figure 26C also shows a case of an object falling from a vehicle. In this case, the VLM also mistook the fallen object for another vehicle. Finally, Figure 26D shows a contradictory situation in which the traffic light turns green, but the vehicle does not start for some reason. The VLM did not handle this contradictory situation well.

**Figure 27:**
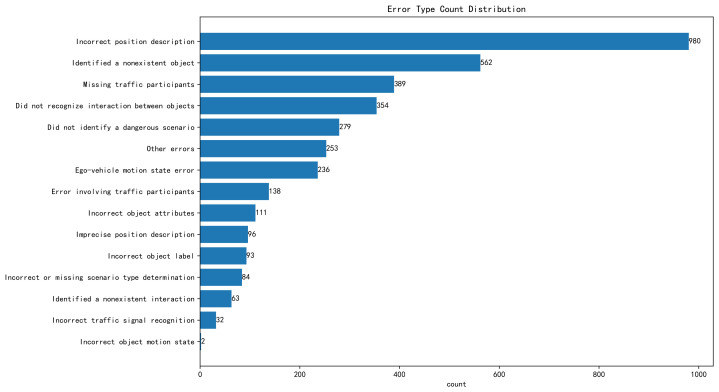
Main error types in RSG generation. "Others" includes other error types, detailed in Figure A3.

**Figure A3:**
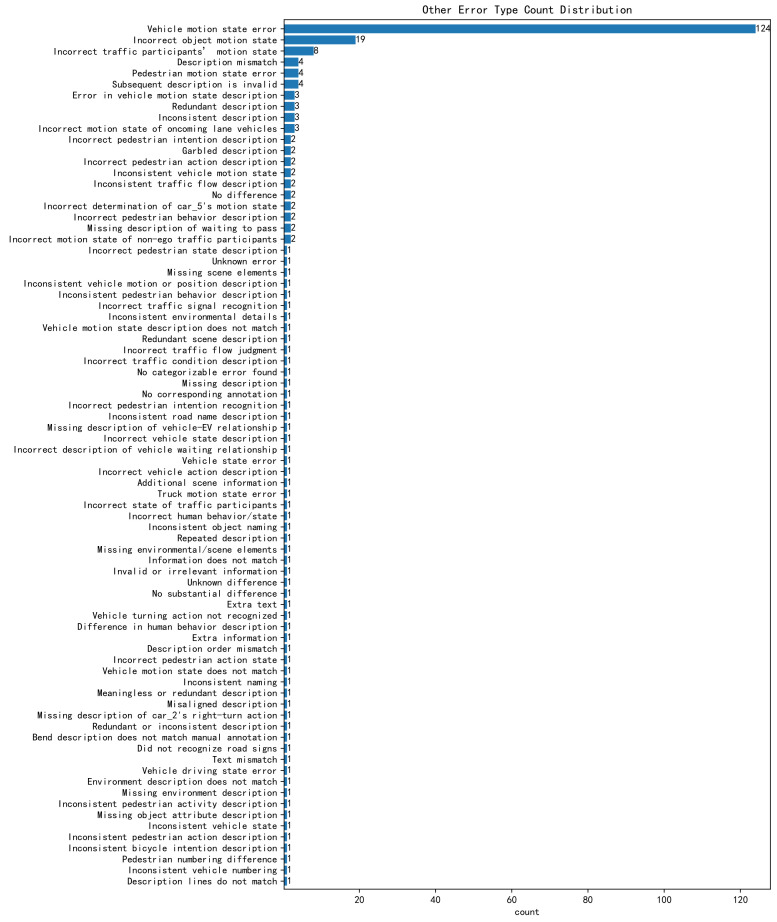
Detailed error types in the “Others” category.

Based on our qualitative analysis, we further examined the error types that arise during RSG generation. As shown in Figure 27, the primary error categories are displayed, whereas Figure A3 details the subtypes classified as “other errors”. In both the main and secondary categories, a long-tail distribution emerges, making it relatively straightforward to identify the principal error sources. A key challenge for VLMs aiming to produce high-accuracy traffic scene descriptions lies in using traffic-centric language to precisely describe object positions (the “Incorrect position description” category). Although seemingly simple, this task involves complex visual reasoning. First, determining the orientation of each object from its ROI region is far from trivial—especially given that the RSG-LLM Benchmark includes complicated lighting conditions (dusk, nighttime), significant object overlap (multiple vehicles mutually occluding one another), objects obscured by environmental elements (e.g., oncoming-lane vehicles blocked by guardrails), and low-resolution frames (particularly common in the NEDO dataset and parts of the Road Hazard Stimuli dataset). Yet identifying an object’s orientation is merely the first step in resolving its detailed location. Next, the model must reason about each individual object’s position relative to the road structure. For example, an intersection can be broken down into near-side or far-side lanes with respect to the ego-vehicle, same-lane vs. opposite-lane positions, as well as entering or exiting roads to the left or right. In more complex intersections, the model must further handle turning bays, auxiliary lanes, roadside parking areas, mid-intersection turns, and even potential traffic violations. These special circumstances appear frequently in the RSG-LLM Benchmark. Moreover, the model might need to make from 10 to 40 such positional judgments in a single scene, which explains why errors in position description stand out as the primary hurdle in RSG generation.

Additionally, “Missing traffic participants” and “Did not identify a dangerous scenario” reflect issues such as object omission, labeling errors, and differences in annotator perspectives during the process of grounding objects in the generated scene. For instance, some annotators—especially in the Road Hazard Stimuli and nuScenes datasets—may focus on the most critical objects while disregarding secondary elements (like vehicles parked far away, oncoming vehicles in the ego-vehicle’s opposite lane, or a large number of cars parked roadside). Meanwhile, a VLM might pay relatively uniform attention to each object in the scene. Furthermore, label overlap, lighting-induced label blurriness, and misrecognitions are all common errors in RSG generation. Another class of errors—“Did not recognize interaction between objects” and “Ego-vehicle motion state error”—stems from motion-related mistakes in spatiotemporal reasoning over image sequences. These errors include, for example, confusing the ego-vehicle’s movement with that of surrounding objects, or incorrectly categorizing a stationary object as moving (e.g., mistaking a parked car for a vehicle traveling in the same direction as the ego-vehicle). Such problems highlight how, despite the current research progress in VLM understanding spatial and semantic relationships within single images, it remains deficient in cross-frame, spatiotemporal feature fusion—an area we plan to address as part of our future work.

Figure 27 and Figure A3 reveal a long-tail distribution of RSG-generation errors, with “Incorrect position description” being particularly critical. Though it seems straightforward, accurately describing object orientation and position requires complex reasoning under poor lighting (e.g., nighttime), overlapping or occluded objects (common in the NEDO and Road Hazard Stimuli datasets), and varied road structures (near-/far-side lanes, turning bays, roadside parking). A single scene can demand dozens of such positional judgments. Other frequent issues, labeled “Missing traffic participants” and “Did not identify a dangerous scenario”, often stem from object omissions, labeling inconsistencies, or annotators focusing on only high-priority objects (e.g., ignoring far-away or opposite-lane vehicles). Label overlap, visibility problems, and misrecognitions also contribute. Finally, “Did not recognize interaction between objects” and “Ego-vehicle motion state error” arise when the model confuses ego-vehicle movement with that of other objects or mislabels parked cars as moving. While VLMs excel at single-image spatial analysis, they lack robust cross-frame, spatiotemporal reasoning—an area we plan to explore further.

### 5.4. Integrating COT into LLM-Based RSG Generation

**Figure 28:**
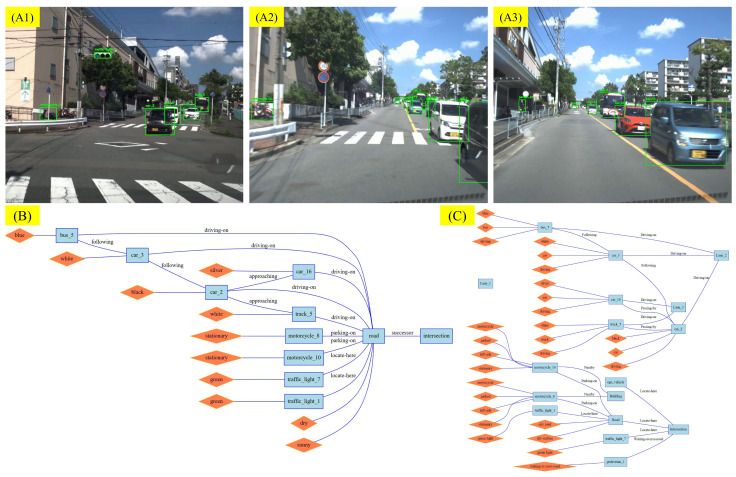
Comparison of GPT-o1 and GPT-4o in the RSG generation task. (**A1**–**A3**) A sample traffic scene from NEDO dataset. (**B**) RSG generated by GPT-4o. (**C**) RSG generated by GPT-o1.

COT (Chain of Thought) [53] is a mature method for improving the performance of LLMs and has been shown to enhance the reasoning ability of LLMs. Similar to LLM Graph Call, COT extends the reasoning process from single-step reasoning to multi-step reasoning. However, different to our LLM Graph Call, COT does not have a fixed reasoning graph structure but automatically determines the next step of reasoning during the reasoning process. The current SOTA COT method is GPT-o1 proposed by OpenAI, which we applied in our experiments. In Figure 28, we show a comparison between GPT-o1 and GPT-4o in the RSG generation task. From the figure, it can be seen that GPT-o1 can recover more environmental information in the RSG generation task, such as road topology and the relationship between objects and specific road elements. However, to control the accuracy of reasoning, this method requires more detailed prior information, such as road topology provided by high-precision maps and the relationship between objects and road elements. In future research, we plan to introduce more prior information into the reasoning process to improve the accuracy of RSG generation.

## 6. Conclusions and Future Work

In this paper, we proposed a semantic traffic scene retrieval method based on LLM-generated Road Scene Graphs (RSGs). By utilizing Visual Large Language Models (VLMs), we effectively transformed raw visual data into RSG structured graph representations, capturing high-level semantic attributes and detailed object relationships. Our method addresses the challenge of retrieving semantically similar traffic scenes from large, heterogeneous datasets and supports multiple query input formats. The proposed RSG-LLM Benchmark dataset provides a valuable resource for evaluating the performance of LLMs in generating RSGs.

Despite the promising results, several challenges remain. First, the accuracy of RSG generation is affected by errors in front-end object detection and label rendering. Future work can focus on integrating more robust object recognition models and improving label rendering techniques to reduce these errors. Second, VLMs sometimes struggle to understand the motion status and spatial relationships in complex traffic scenes. Introducing more prior information, such as object kinematic data from LiDAR or depth models, may enhance the reasoning ability of VLMs. Additionally, exploring advanced reasoning methods such as Chain-of-Thought (CoT) prompting may further improve the performance of RSG generation. Finally, extending the RSG-LLM Benchmark dataset to include more diverse and complex traffic scenes will provide a more comprehensive evaluation framework and promote further research in this field.

Currently, due to the computational limitations of large models, our model cannot achieve real-time processing. However, we hope that this research will serve as a foundation for LLM-assisted autonomous driving and provide a new direction for future research. For example, implementing Retrieval Argumented Generation (RAG) on autonomous driving systems and searching for similar scenes in the scene library based on the currently perceived scene during vehicle driving could assist vehicle decision making and path generation. We will continue expanding our research on LLMs in the field of traffic scene understanding, exploring more application scenarios, and proposing solutions.

## Figures and Tables

**Table 1:** Extensible scenario description and attribute value ranges.

Attribute	Values
Weather	Sunny, Rain, Snow, Fog, Sandstorm, Haze
Lighting	Day, Night, Dusk, Dawn, Strong light, Glare, Tunnel light change
Road type	Highway, Urban road, Rural road, Mountain road, Unpaved road, Intersection, Bridge, Tunnel
Traffic	Traffic jam, Clear road, Peak time, Off-peak, Heavy traffic, Light traffic
Environment	Urban area, Suburbs, Countryside, Industrial zone, Residential area, Commercial area, School zone, Near hospital
Special scenarios	Construction zone, Accident site, Emergency vehicle, Road closure, Detour, Special event
Road condition	Good road, Potholes, Waterlogged, Snow-covered, Icy, Under construction, Lane markings clarity
Driving behavior	Overtaking, Lane change, U-turn, Highway merge, Exit highway, Parking, Start
Special participants	Disabled, Children, Elderly, Emergency vehicles
Unusual events	Sudden accident, Road obstacle, Sudden braking ahead
Visual interference	Strong sunlight, Reflection, Raindrops blocking lens, Snow blocking lens, Blinding light
Speed and state	High speed, Low speed, Stopped, Accelerating, Decelerating
Multimodal interaction	Traffic police, Construction worker directing traffic
Terrain	Steep slope, Sharp turn, Uneven road
Complex traffic	Multi-lane change, Complex intersection, Overpass, Roundabout, Tunnel, Bridge

**Table 2:** Examples of objects and relationships in an RSG.

Object 1	Object 2	Relationship
Vehicle	Lane	driving-on, waiting-on, parking-on
Vehicle	Road	driving-on, waiting-on, parking-on
Vehicle	Intersection	driving-on, stop-behind, turn-left, turn-right, go-straight
Vehicle	Pedestrian	on-same-lane, following, approaching, waiting-at-crossroad, waiting-for, behind, passing-by, overtaking
Vehicle	Vehicle	following, overtaking, passing-by, waiting-at-crossroad, waiting-for, behind, may-intersect
Vehicle	Barrier	passing-by, avoiding
Pedestrian	Lane	nearby, walking-along
Pedestrian	Road	nearby, walking-along
Pedestrian	Intersection	nearby, crossing
Pedestrian	Pedestrian	nearby
Pedestrian	Vehicle	behind, waiting-at-crossroad, may-intersect
Pedestrian	Barrier	behind
Barrier	Lane	locate-here
Barrier	Road	locate-here
Barrier	Intersection	locate-here
Barrier	Pedestrian	nearby
Barrier	Vehicle	passing-by, avoiding
Road	Lane	predecessor, successor
Road	Intersection	predecessor, successor
Intersection	Lane	predecessor, successor
Intersection	Road	predecessor, successor

**Table 3:** Statistics of the RSG-LLM benchmark dataset.

Data Source	Videos	Number of RSG Graphs	Video Length	Video Resolution	Object Recognition Source	Recording Location	Special Notes
NEDO	400	400	6 s	Variable (e.g., 1368 × 1096)	YOLO	Japan, Nagoya	Contains Japan-specific traffic signs; roads are typically narrow
nuScenes	300	300	6 s ^*^	1600 × 900	Manual Annotation	Boston, Singapore	Contains very accurate object location information
Road Hazard Stimuli	300	300	7 s	1280 × 720	YOLO	Boston	Contains numerous traffic accident scenes and similar non-hazardous scenes

**Note:** ^*^ nuScenes video clips are typically 20 s in length. Here, we only use the first 6 s.

**Figure A2:**
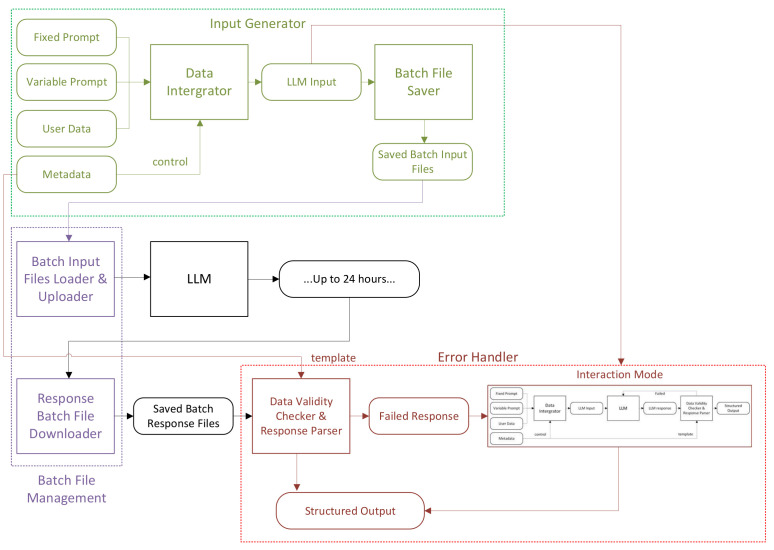
Node implementation in the LLM Call Graph with Batch mode.

## Data Availability

The RSG-LLM Benchmark, as well as the framework we used to manage VLM interaction, can be found at the following link: https://github.com/TianYafu/RSG-LLM/tree/main, accessed on 3 April 2025.

## Appendix A. LLM Call Graph

The engineering implementation of a generic node is illustrated in Figure A1. For clarity, we have separated User Data from the Variable Prompt in the figure. User Data mainly consists of video or image data, while the Variable Prompt primarily handles textual data passed from upstream nodes. The Metadata in the diagram defines how the node combines the data, which is essentially the specific implementation of the connection function $f_{i}$. A generic node consists of the following three main components: the Input Generator, LLM Call, and Error Handler. The Input Generator is responsible for combining the System Prompt, video or image data, and content generated by upstream nodes to create the input for the LLM. The LLM call handles the execution of the LLMs and passes the output to downstream nodes. The LLMs here could be any large language models such as GPT-4 or Claude. Lastly, the Error Handler manages errors that occur during LLM calls, such as empty return values or invalid output formats (in the case of generating JSON). Along with the prompt, a corresponding JSON schema is provided to automatically validate whether the LLM output is in the correct format.

Some LLM providers offer a Batch API to reduce costs. Batch API is an asynchronous task processing method where users can submit tasks in batches to the server, which processes them during idle times. This approach allows researchers to significantly save on costs. In Figure A2, the node implementation is modified to use Batch API. Compared to the sequential execution of nodes in Figure A1, Batch API introduces a file management feature to handle the batched tasks. In contrast to sequential execution, the error handling module in batch processing cannot immediately validate the output and retry in case of errors. Therefore, after the batch task is completed, we add an independent validation module to check the validity of the output and fix any errors.

## Appendix B. Minor Errors in RSG Generation

Since the error rate in the RSG generation process follows a long-tail distribution, the minor error types and their quantities are shown in Figure A3.
